# Recent Advances in Carbon Cathode Materials toward High‐Performing Zinc‐Ion Hybrid Supercapacitors

**DOI:** 10.1002/advs.76289

**Published:** 2026-06-28

**Authors:** Take Hirooka, Zhaolin Liu, Ivan P. Parkin, Guanjie He

**Affiliations:** ^1^ Department of Chemistry University College London London UK; ^2^ Institute of Materials Research and Engineering (IMRE) A*STAR (Agency for Science, Technology and Research) Singapore

**Keywords:** carbon cathode materials, charge‐storage mechanisms, heteroatom doping, porous carbon, pore engineering, zinc‐ion hybrid supercapacitors

## Abstract

Aqueous zinc‐ion hybrid supercapacitors (ZHSCs) have emerged as promising next‐generation electrochemical energy‐storage devices due to their intrinsic safety, low cost, and ability to combine the high energy density of batteries with the high power density and long lifespan of supercapacitors. However, development is still in its infancy, and challenges include the limited performance of the capacitive/pseudocapacitive cathode material, whose structure and chemistry largely determine the device's energy‐storage capabilities. Carbon materials, particularly porous carbons, have been extensively studied as cathode materials in ZHSCs. Recent advancements—including activated carbons, template‐assisted carbons, metal‐organic‐framework‐derived carbons, and biomass‐derived carbons—have enabled significant progress in tailoring pore structures, chemical functionalities, and electronic properties to enhance zinc‐ion energy storage. This review summarizes these developments, examining their synthesis methods and the design principles shaping their electrochemical behavior. Additionally, strategies of heteroatom doping and hybridization were critically evaluated, highlighting pathways to enhance electronic/ionic conductivities and pseudocapacitance. Finally, remaining challenges—including limited tunability of pore structures, incomplete mechanistic understanding, and restricted performance—were outlined, along with future directions for rational design and scalable synthesis. This review offers an updated perspective on engineering carbon cathode materials for high‐performing ZHSCs and their potential in advancing safe, sustainable electrochemical energy storage.

## Graphical abstract

Aqueous zinc‐ion hybrid supercapacitors (ZHSCs) have emerged as promising next‐generation electrochemical energy‐storage devices. Porous carbon materials have been extensively studied as cathodes, with advances in tailoring pore structures and material properties to enhance performance. This review examines these developments and related modification strategies, offering an updated perspective on engineering carbon cathode materials for ZHSCs.

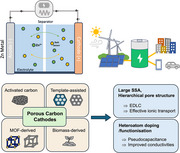

## 1 Introduction

**FIGURE 1:**
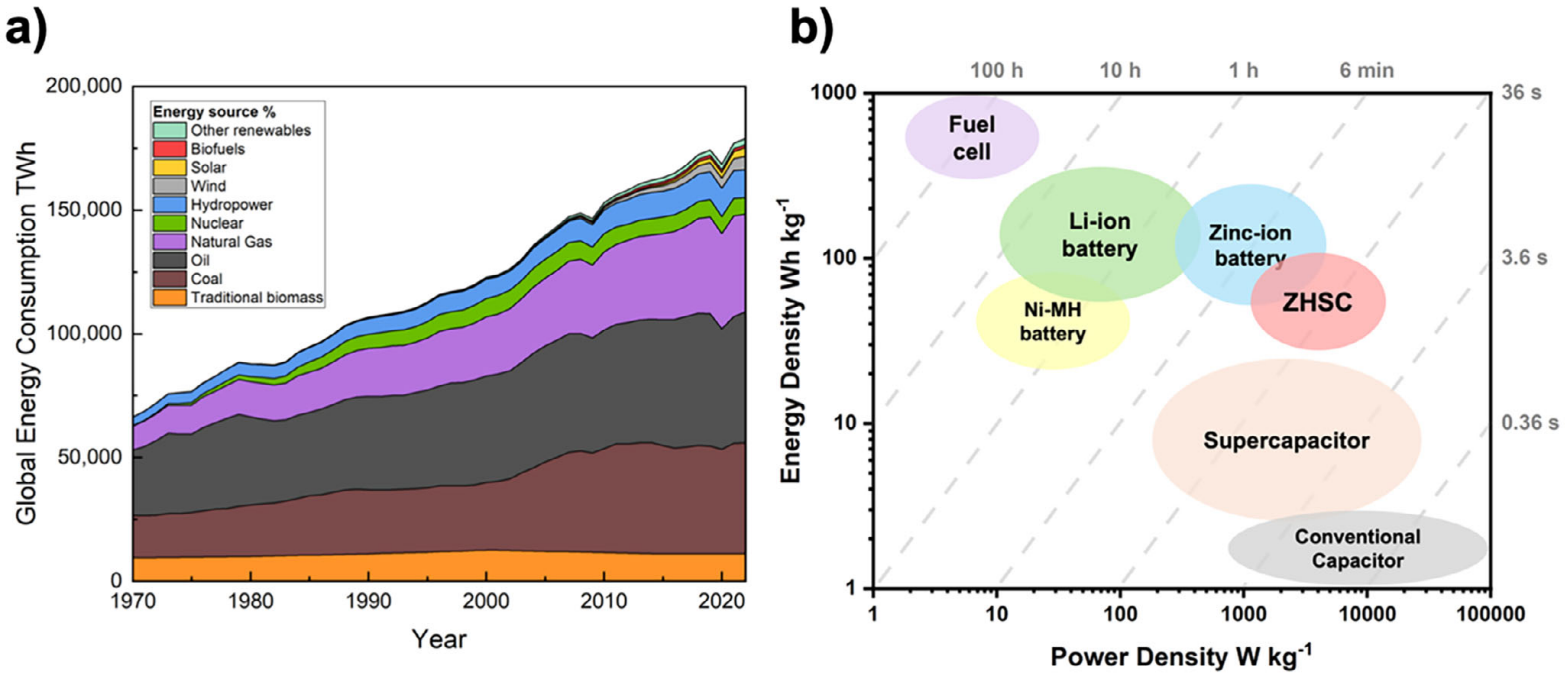
(a) Global energy consumption by source. Adapted under terms of the CC‐BY 4.0 license [1]. Copyright 2024, The Authors, published by Our World in Data. (b) Ragone plot of various EES devices.

The overreliance and excessive consumption of non‐renewable fossil fuels have led to severe environmental pollution, pressing the demand for alternative renewable energy sources such as solar and wind power (Figure 1a) [1]. However, these energy sources are commonly intermittent and inconsistent, requiring established energy storage for efficient use. Meanwhile, with advancements and popularization of technologies such as EVs, portable and wearable electronics, and smart grids, considerable attention is being directed toward developing high‐performing and long‐lasting energy storage devices [2, 3]. In particular, electrochemical energy storage (EES) devices, such as batteries and supercapacitors, have been widely researched and adopted in commercial applications [4, 5].

Among them, rechargeable lithium‐ion batteries have achieved significant success [6]. However, the use of flammable organic electrolytes and the scarcity of lithium have raised issues of safety and high cost, respectively, shifting interest toward alternative metal ion chemistries for more sustainable EES. The use of zinc is highly promising due to its suitable redox potential (−0.76 V vs. standard hydrogen electrode), high theoretical and volumetric capacities (820 mA h g^−1^ and 5851 mA h cm^−3^), and low toxicity. Zinc is compatible with non‐flammable aqueous electrolytes, enhancing their safety and environmental friendliness, while their high abundance (≈300x of lithium) makes them low‐cost. These collective advantages make aqueous zinc‐based energy storage a favorable system for next‐generation EES devices [7, 8].

Thus far, primary attention has been given to aqueous zinc‐ion batteries (ZIBs), which follow working principles analogous to LIBs. The reversible storage of zinc ions through intercalation‐type redox reactions at the cathode (commonly metal oxides such as manganese and vanadium oxides), matched with fast zinc ion stripping/deposition at the zinc metal anode (Zn ↔ Zn^2+^), demonstrates large energy densities (≈150 W h kg^−1^) for ZIBs [8]. However, sluggish reaction kinetics and harsh volume changes associated with the intercalation‐based cathode can cause low power densities and short cycling lives, limiting their practical applications [9, 10]. Consequently, the emergence of zinc‐ion hybrid supercapacitors (ZHSC) has gained considerable attention, combining the design principles of ZIBs with those of supercapacitors (SCs). SCs employ two types of surface‐controlled charge storage mechanisms, namely electrochemical double‐layer capacitance (EDLC) and pseudocapacitance [11]. EDLC stores charge through physical adsorption/desorption of ions, whereas pseudocapacitance stores charge via rapid Faradaic reactions on electrode surfaces. These stable and fast processes grant SCs with high‐power densities (> 300 W kg^−1^) and long cycling lifetimes (> 100 000), but their surface‐restricted mechanisms limit energy densities, which are insufficient for many applications [12, 13]. By adopting the ZIB configuration and substituting one electrode with an SC‐type (capacitive/pseudocapacitive) electrode, a ZHSC can be assembled, combining the high energy density typical of ZIBs with the advantages of high power density and long cycling lifetime characteristic of conventional SCs. This hybrid of battery and SC technology marks ZHSCs uniquely within the Ragone plot (Figure 1b), capable of addressing the increasing demand for both high energy and high power densities, thus opening promising avenues for widespread application [14, 15].

**FIGURE 2:**
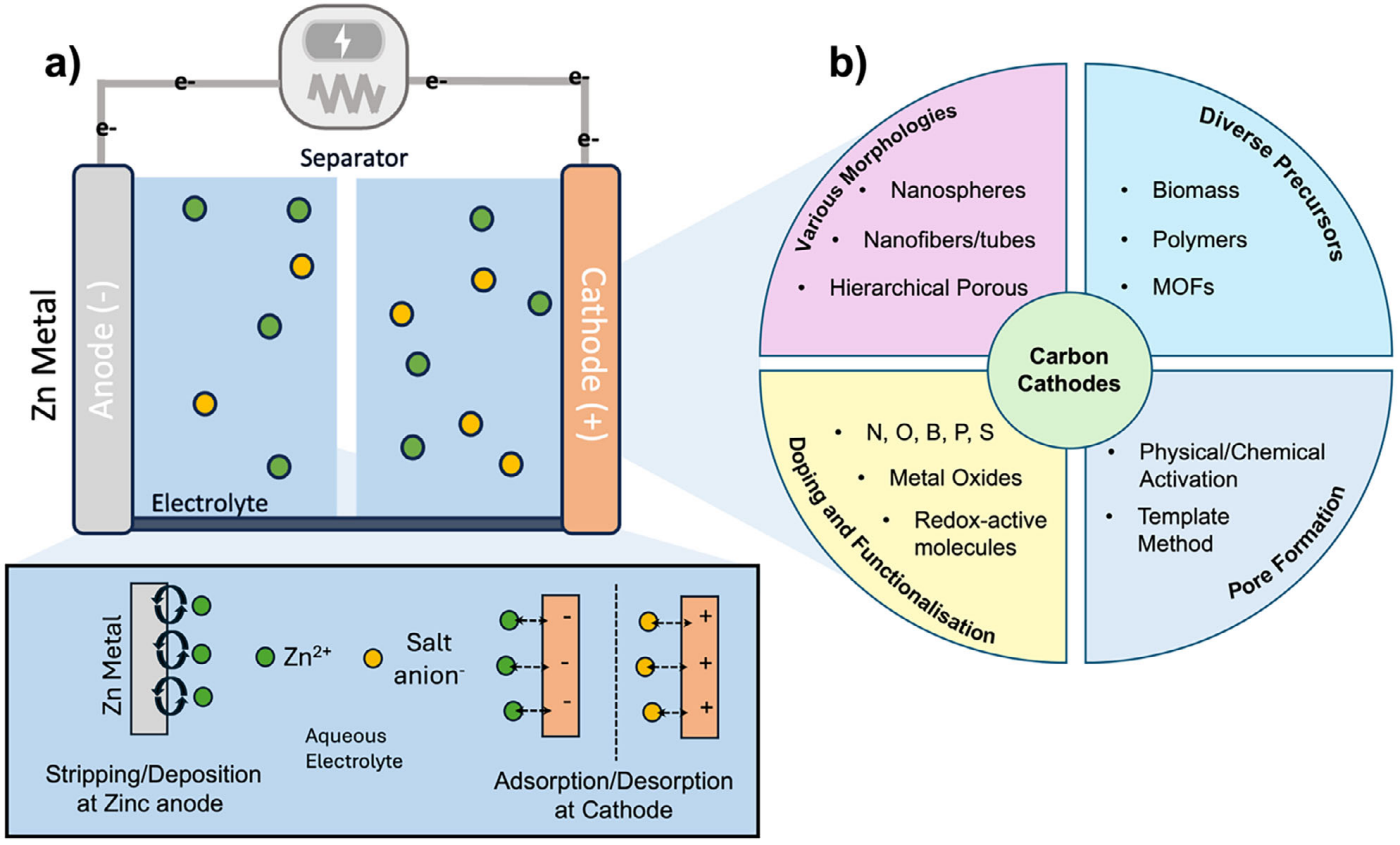
(a) Schematic representation of the ZHSC setup with SC‐type cathode and zinc metal anode, with charge storage processes. (b) Summary of the aspects of carbon cathode materials for ZHSCs.

ZHSCs are still a relatively new technology, and their development is still in its infancy. In a conventional setup, metallic zinc is commonly used as the battery‐type anode, aqueous zinc salts serve as the electrolyte, and a capacitive/pseudocapacitive material is used for the SC‐ type cathode (Figure 2a). Although the alternative setup with an SC‐type anode and a battery‐type cathode can be employed, the direct use of zinc metal foils/powders as the anode is generally preferred due to zinc's attractive properties, such as high theoretical capacities, fast mechanisms (stripping/deposition), low cost, and sustainability, as previously mentioned. Given the superior capacity performance of zinc anodes, the primary performance limit for ZHSCs then becomes the SC‐type cathode material, which currently cannot reach the high energy densities of ZIBs (battery‐type cathode) [16]. Furthermore, questions regarding the ions involved in the charge storage mechanisms at the SC‐type cathode in ZHSCs remain unclear, calling for a deeper understanding. Thus, the development and research of SC‐type electrode materials with large capacitances and fast kinetics, along with mechanistic studies, is essential to understanding and realizing the full potential of ZHSCs.

Currently, carbon materials have been extensively studied. Owing to their good electrical conductivity, high mechanical and chemical stability, and versatile structures, carbon materials are widely explored as active electrode materials in SCs and ZHSCs [17, 18, 19, 20]. Particularly, porous carbons with high specific surface areas (SSAs) and well‐developed pore structures can provide extensive electrode‐electrolyte contact, creating numerous electrochemically active sites and facilitating rapid ion and electron transfer through the material. These properties are essential for enhancing the capacity and power densities of the electrode, making porous carbon materials highly desirable for ZHSCs. Additionally, modification strategies such as heteroatom doping and hybridization with redox‐active materials can be applied to further enhance electrode performance. It is evident that, with appropriate design and tailored construction, effective carbon cathode materials can be developed for ZHSCs, offering promise toward practical commercialization.

This review provides an up‐to‐date, comprehensive overview of advancements in carbon cathode materials for ZHSCs, with a primary focus on porous carbons, encompassing developments in activated carbons (ACs), template‐assisted carbons, metal‐organic framework‐derived carbons, and biomass‐derived carbons. Since the first report of ZHSCs in 2018 [21], rapid growth in research on carbon‐based cathode materials has led to significant performance improvements. In particular, this review focuses on key developments over the past 3–4 years, during which major advances in the structural engineering and functionalization of diverse porous carbons have been made, distinguishing recent work from earlier studies that largely focused on conventional ACs with limited structural control. Unlike previous reviews that examine these carbon material classes separately, this work offers a unified and comparative perspective, establishing structure‐property relationships across the full range of porous‐carbon materials. Their synthesis methods, physicochemical properties, and electrochemical performances are assessed, highlighting how pore hierarchy, dopant chemistry, and morphology influence ion storage and transport. Additionally, approaches to improve the performance of carbon cathodes, such as heteroatom doping and material hybridization, are critically analyzed. Finally, an overview and outlook are provided, emphasizing remaining challenges and future research directions necessary for the rational development of next‐generation carbon cathode materials for ZHSCs.

## 2 Carbon Cathode Materials

In a typical ZHSC setup, the cathode is generally the SC‐type electrode, dependent on its materials’ capacitive and/or pseudocapacitive properties. Carbon, with its good electrical conductivity, excellent stability, tunable structures, and abundant raw materials, has become the most commonly used electrode material for SCs and ZHSCs. Notably, porous carbon materials have attracted considerable attention due to their large SSA and controllable pore sizes. A large SSA can enable extended electrode‐electrolyte contact, increasing the number of accessible active sites and thereby enlarging the electrode's energy storage capabilities. Meanwhile, well‐developed pore structures can support the efficient transport of ions throughout the electrode, allowing for greater power performance. Additional modifications to the carbon material, such as doping and functionalization, can further enhance properties and boost performance. Thus, a diverse range of porous carbons with various synthesis methods has been recently reported and tested as active cathode materials for ZHSCs. A summary of the performance of porous carbon cathode materials is shown in Table 1. It should be noted that electrochemical performance metrics reported in the literature are often not directly comparable due to variations in experimental conditions. Therefore, caution is required when comparing results across studies.

**TABLE 1:** Performance summary of reported ZHSCs with porous carbon cathode materials.

Cathode Material Class	Material name	Electrolyte	Potential Window V	Capacity/capacitance	Peak energy density W h kg^−1^	Peak power density kW kg^−1^ / Corresponding energy density W h kg^−1^	Cycling stability % / Cycling number	Refs.
AC	AC (commercial)	2 M ZnSO_4_	0.2 – 1.8	121 mA h g^−1^ (0.1 A g^−1^)	84	14.9 / 30	91% / 10 000 (1 A g^−1^)	[21]
	MSAC‐1	2 M ZnSO_4_	0.3 – 1.8	176 mA h g^−1^ (0.5 A g^−1^)	188	10.666 / 77	78% / 40 000 (10 A g^−1^)	[22]
	HPAC	3 M Zn(CF_3_SO_3_)_2_	0.01 – 1.8	231 mA h g^−1^ (0.5 A g^−1^)	—	11.4 / 77.5	70% / 18 000 (10 A g^−1^)	[23]
	RbPC	1 M Zn(CF_3_SO_3_)_2_	0.2 – 1.8	247.6 mA h g^−1^ (1 A g^−1^)	178.2	72.3 / 52.2	99.8% / 20 000 (10 A g^−1^)	[24]
Template‐assisted porous carbon (Hard template)	MCHS	2 M ZnSO_4_	0.2 – 1.8	174.7 mA h g^−1^ (0.1 A g^−1^)	129.3	13.7 / 36.8	100% / 10 000 (1 A g^−1^)	[25]
	HBC	2 M ZnSO_4_	0.2 – 1.8	95.4 mA h g^−1^ (0.1 A g^−1^)	—	—	> 100% / 1000 (1 A g^−1^)	[26]
	HCS_t6_	1 M ZnSO_4_	0.2 – 1.8	260 F g^−1^ (0.1 A g^−1^)	85.3	16 / ‐	99.3% / 30 000 (2 A g^−1^)	[27]
	N‐HCNbs‐6	2 M ZnSO_4_	0.2 – 1.8	136.2 mA h g^−1^ (0.2 A g^−1^)	109	40 / 53	96% / 24 000 (5 A g^−1^)	[28]
	HHPC	2 M ZnSO_4_	0.2 – 1.8	147 mA h g^−1^ (0.2 A g^−1^)	117.6	40 / 56.7	88% / 20 000 (5 A g^−1^)	[29]
	HT‐NMPC‐1/1	1 M Zn(CF_3_SO_3_)_2_	0.2 – 1.8	146.65 mA h g^−1^ (0.2 A g^−1^)	121.5	8 / 75.7	93.09% / 8000 (2 A g^−1^)	[30]
	HNPC	1 M ZnSO_4_	0 – 1.8	177.8 mA h g^−1^ (4.2 A g^−1^)	107.3	24.9 / ‐	99.7% / 20 000 (16.7 A g^−1^)	[31]
	NPC‐5	1 M ZnSO_4_	0.2 – 1.8	115.44 mA h g^−1^ (0.1 A g^−1^)	85.3	—	94.5% / 10 000 (200 mV s^−1^)	[32]
	LPC‐800	1 M ZnSO_4_	0.2 – 1.8	157 F g^−1^ (0.1 A g^−1^)	—	—	87% / 20 000 (2 A g^−1^)	[33]
Template‐assisted porous carbon (Soft template)	HD‐HPC	2 M ZnSO_4_	0.2 – 1.8	169 F g^−1^, 50.7 F cm^−3^ (0.1 A g^−1^)	18 W h L^−1^	—	≈100% / 8000 (10 A g^−1^)	[34]
	900 N‐mgc	2 M Zn(CF_3_SO_3_)_2_	0 – 1.8	43 F g^−1^ (0.2 A g^−1^)	19.4	—	80% / 3000 (2 A g^−1^)	[35]
	NPC‐2	2 M ZnSO_4_	0.2 – 1.7	166.5 mA h g^−1^ (0.1 A g^−1^)	124	7.5 / 52.5	82.4% / 5000 (5 A g^−1^)	[36]
	N‐HNCS‐1	2 M ZnSO_4_	0.2 – 1.8	142 mA h g^−1^ (0.5 A g^−1^)	115.4	—	98% / 10 000 (10 A g^−1^)	[37]
	CS‐750	3 M Zn(CF_3_SO_3_)_2_	0 – 1.8	262.8 mA h g^−1^ (0.2 A g^−1^)	160.8	—	93.2% / 200 000 (20 A g^−1^)	[38]
Template ‐assisted porous carbon (other template)	AHPCN‐29	2 M ZnSO_4_	0.2 – 1.8	157.1 mA h g^−1^ (0.1 A g^−1^)	125.7	16 / 88.3	100% / 10 000 (5 A g^−1^)	[39]
	N‐CNAG	1 M ZnSO_4_	0 – 2	706 F g^−1^ (1 A g^−1^)	392	5 / 196	87.4% / 10 000 (10 A g^−1^)	[40]
	PCC‐3	1 M Zn(CF_3_SO_3_)_2_	0.2 – 1.8	345 F g^−1^ (0.5 A g^−1^)	119.02	13.238 / 57.31	99% / 10 000 (5 A g^−1^)	[41]
MOF‐derived porous carbon	MPC‐2	3 M Zn(CF_3_SO_3_)_2_	0 – 1.8	289.2 F g^−1^ (0.2 A g^−1^)	130.1	7.8 / 59	96.7% / 10 000 (10 A g^−1^)	[42]
	A‐NOCNS	2 M Zn(CF_3_SO_3_)_2_	0 – 1.9	176.48 mA h g^−1^ (0.2 A g^−1^)	162.88	28.43 / 58.54	90.23% / 20 000 (10 A g^−1^)	[43]
	ZIF‐8‐800(KOH)	ZnSO_4_	0.2 – 1.8	130 mA h g^−1^ (0.1 A g^−1^)	125	3.938 / 71	77.8% / 9000 (1 A g^−1^)	[44]
	ZMDPC‐700	2 M ZnSO_4_	0.2 – 1.8	255.6 F g^−1^ (0.5 A g^−1^)	90.88	4.5 / 57.71	97.8% / 10 000 (10 A g^−1^)	[45]
	HPNCF	2 M ZnSO_4_	0.2 – 1.8	240 F g^−1^ (1 A g^−1^)	80.1	—	99.58% / 50 000 (10 A g^−1^)	[46]
Biomass‐derived carbon	LDC3	LDH3 (hydrogel)	0 – 2	229 mA h g^−1^ (0.5 A g^−1^)	226	7.515 / 65	≈100% / 30 000 (5 A g^−1^)	[47]
	HPC	2 M ZnSO_4_ + 1 M Na_2_SO_4_	0.01 – 1.8	305 mA h g^−1^ (0.1 A g^−1^)	118	3.1582 / 63.7	94.9% / 20 000 (2 A g^−1^)	[48]
	R‐NC‐K2	2 M ZnSO_4_	0.2 – 1.8	212 mA h g^−1^ (0.2 A g^−1^)	168	3.7 / ‐	91% / 50 000 (10 A g^−1^)	[49]
	LLC‐5	2 M ZnSO_4_	0.1 – 1.8	164.3 F g^−1^ (0.2 A g^−1^)	65.2	13.3 / 23.3	96.6% / 10 000 (1 A g^−1^)	[50]
	WPAC‐3	2 M Zn(CF_3_SO_3_)_2_	0 – 1.8	335.9 F g^−1^ (0.5 A g^−1^)	151.3	18 / 82.5	97.4% / 25 000 (10 A g^−1^)	[51]
	CSK	2 M ZnSO_4_	0 – 1.8	192 mA h g^−1^ (0.5 A g^−1^)	172	9 / 90	90% / 50 000 (5 A g^−1^)	[52]
	MGC_3_‐800	ZnSO_4_ gel	0 – 1.8	186.1 mA h g^−1^ (0.2 A g^−1^)	167.5	6.3 / 58.2	92.6% / 10 000 (10 A g^−1^)	[53]
	GA	1.5 M ZnSO_4_	0 – 1.8	353.1 F g^−1^ (0.1 A g^−1^)	158.9	14.8 / 77.2	84.2% / 10 000 (10 A g^−1^)	[54]
	LPC‐1‐3	1 M ZnSO_4_	0.2 – 1.8	279 F g^−1^ (0.1 A g^−1^)	99.1	80.1 / ‐	93% / 10 000 (1 A g^−1^)	[55]
	TFMA	2 M ZnSO_4_	0.1 – 1.8	107 mA h g^−1^ (1 A g^−1^)	110.8	5.52 / 36.8	≈100% / 8000 (5 A g^−1^)	[56]
	JC	2 M ZnSO_4_	0.2 – 1.8	204 F g^−1^ (0.5 A g^−1^)	73	3.2 / 43	130% / 20 000 (10 A g^−1^)	[57]
	ASICKOH	1 M ZnSO_4_	0.1 – 1.45	208 F g^−1^ (0.1 A g^−1^)	—	—	84.5% / 10 000 (5 A g^−1^)	[58]
	SFPC‐A13	2 M ZnSO_4_	0 – 1.9	221.1 mA h g^−1^ (0.5 A g^−1^)	160.9	9.473 / 30	100% / 175 000 (10 A g^−1^)	[59]
	BP‐H_3_PO_4_	2 M ZnSO_4_	0 – 2	221.05 F g^−1^ (0.1 A g^−1^)	120	5.502 / 18.37	83% / 50 000 (1 A g^−1^)	[60]
	PC‐NaOH/Na_2_SO_3_‐7	2 M ZnSO_4_	0.2 – 1.8	362 F g^−1^ (0.1 A g^−1^)	128.62	16 / 52.89	99.3% / 120 000 (7 A g^−1^)	[61]
	PFPC‐A14	2 M ZnSO_4_	0 – 1.8	192.2 mA h g^−1^ (0.5 A g^−1^)	172.9	8.998 / 31.9	96.7% / 30 000 (10 A g^−1^)	[62]
Heteroatom‐doped carbon	OPCNF‐20	1 M ZnSO_4_	0.2 – 1.8	136.4 mA h g^−1^ (0.1 A g^−1^)	97.74	9.92 / ‐	81% / 50 000 (5 A g^−1^)	[63]
	Zn‐MET‐800	2 M ZnSO_4_	0.1 – 1.7	164.2 mA h g^−1^ (0.1 A g^−1^)	128.5	4.7 / ‐	90.3% / 30 000 (10 A g^−1^)	[64]
	CG2	1 M ZnSO_4_	0.2 – 1.8	241.1 mA h g^−1^ (0.1 A g^−1^)	191.6	12.684 / 84.6	81.87% / 20 000 (5 A g^−1^)	[65]
	N, P‐OLC	2 M ZnSO_4_	0.2 – 1.8	420.3 F g^−1^ (0.5 A g^−1^)	149.5	26.7 / ‐	77.8% / 50 000 (10 mA cm^−2^)	[66]
	SNPC‐800	2 M ZnSO_4_ + 0.5 M ZnCl_2_	0.1 – 1.8	179.1 mA h g^−1^ (0.1 A g^−1^)	89.6	1.9976 / 28.3	99.8% / 5000 (1 A g^−1^)	[67]
	S, N‐CNC	2 M ZnSO_4_	0 – 1.8	165.5 mA h g^−1^ (1 A g^−1^)	148.9	7.2 / 60.6	70% / 10 000 (5 A g^−1^)	[68]
	P&B‐AC	2 M ZnSO_4_	0.2 – 1.8	169.4 mA h g^−1^ (0.5 A g^−1^)	169.4	20 / 66.7	88% / 30 000 (10 A g^−1^)	[69]
	CPTHB‐B2	2 M ZnSO_4_	0.2 – 1.8	411.5 F g^−1^ (0.5 A g^−1^)	131.9	42.1 / 62.4	98.5% / 10 000 (10 A g^−1^)	[70]
	BNC	2 M ZnSO_4_	0.05 – 1.8	204 mA h g^−1^ (0.2 A g^−1^)	178.7	17.5 / 115.4	97% / 40 000 (10 A g^−1^)	[71]
	N‐S‐ONC	2 M ZnSO_4_	0 – 1.8	111 F g^−1^ (0.5 A g^−1^)	50	3.6 / 22	73% / 50 000 (5 A g^−1^)	[72]
	APN	3 M Zn(CF_3_SO_3_)_2_	0 – 1.8	138 mA h g^−1^ (0.5 A g^−1^)	136	—	94% / 10 000 (1 A g^−1^)	[73]

### 2.1 Activated Carbon

Activated carbon (AC) have been widely explored as electrode materials in SCs due to their significant SSA, abundant pores, low cost, and ease of manufacturing [74]. ACs are a type of porous carbon produced through a straightforward and efficient method involving carbonization and activation steps. Carbonization refers to the production of carbonaceous materials, typically through high‐temperature pyrolysis of carbon‐rich precursors in an inert atmosphere. Novel techniques, such as hydrothermal carbonization [75], microwave‐assisted carbonization [76], and laser‐assisted carbonization [77], can also be employed. The following activation is the pore‐forming step, which creates pores in the carbon materials through chemical or physical means. Activation involves the pyrolysis of carbon materials in the presence of an activation agent, where reactions with the agent consume carbon atoms throughout the material to form pores. In the case of physical activation, gases such as steam and CO_2_ are used [78], whereas chemical activation employs chemicals like alkali metal hydroxides, alkali metal carbonates, and phosphoric acids [79, 80, 81]. The simple technique provides ACs with very high SSA, ranging from 500 to 3500 m^2^ g^−1^. Additionally, controlling the temperature, heating times, and choice of activation agent enables modulation of pore sizes and structures [82], making ACs a suitable option for ZHSCs.

**FIGURE 3:**
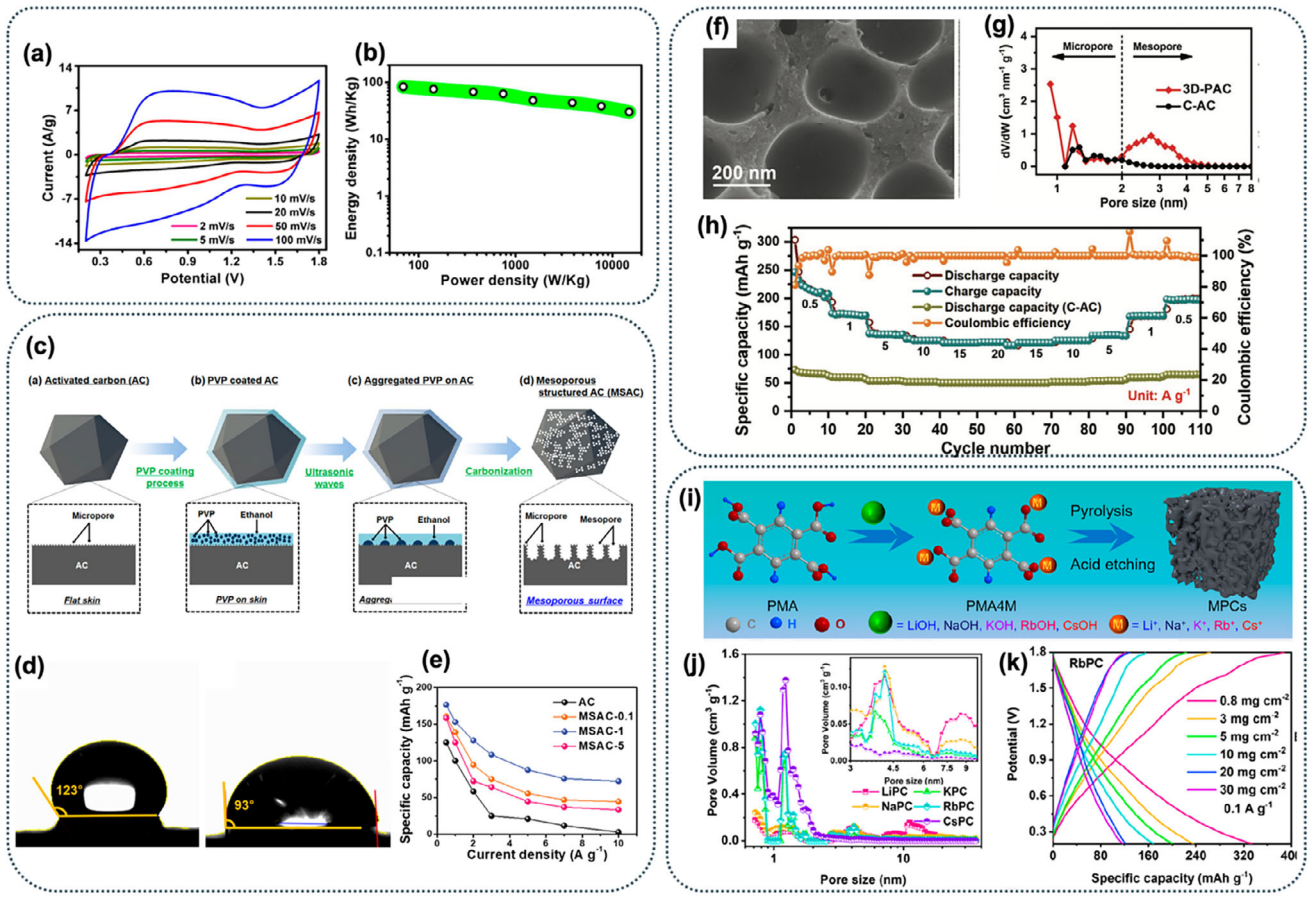
(a) CV curves of ZHSC at 2 – 100 mV s^−1^. (b) Ragone plot of ZHSC. Reproduced with permission [21]. Copyright 2018, Elsevier. (c) Fabrication process of MSAC. (d) electrolyte‐electrode contact angle of AC (left) and MSAC‐1 (right). (e) Calculated specific capacity at a range of current densities (0.5 – 10 A g^−1^). Reproduced with permission [22]. Copyright 2020, Elsevier. (f) SEM image of HPAC. (g) Calculated PSD of HPAC from N_2_ adsorption/desorption isotherms. (h) Rate capabilities of HPAC‐based ZHSC. Reproduced with permission [23]. Copyright 2020, Wiley‐VCH. (i) Fabrication process of MPCs. (j) Calculated PSD of MPCs. (k) GCD curves of RuPC‐based ZHSCs at different mass loadings. Reproduced with permission [24]. Copyright 2022, American Chemical Society.

Early research success of ZHSCs included the use of commercial AC as the SC‐type cathode material [21]. Matched with a zinc metal foil anode and 2 m zinc sulphate (ZnSO_4_) electrolyte, the cyclic voltammetry (CV) curves exhibited a quasi‐rectangular shape (Figure 3a), differing from ZIB or symmetric SC configurations, indicating a hybrid energy storage mechanism. The assembled cell achieved a high energy density of 84 W h kg^−1^, good power densities of 14.9 kW kg^−1^ (Figure 3b), and excellent cycling stability (91% capacity retention after 10 000 cycles). The researchers suggested that the large specific surface area (1923 m^2^ g^−1^) of the AC was responsible for the high energy density, proposing that ion adsorption/desorption was the primary charge storage mechanism at the cathode, while zinc deposition/stripping occurred at the anode. These initial findings proved to be a breakthrough for ZHSCs, sparking interest in them within the field of aqueous zinc‐based energy storage.

Due to their low cost and the existing large‐scale production, commercial ACs (typically physically activated) have continued to be used as cathode materials for investigating various properties of ZHSCs. However, the dominant microporous nature (pore size < 2 nm) of traditional ACs can impede the electrode's ability for efficient ion diffusion into the bulk of the porous structure, limiting their capacities and rate performances. Conversely, incorporating mesopores (pore size 2–50 nm) in ACs has shown considerable success, allowing for wider tunnels and supporting shorter ion transfer pathways, thereby improving the cathode's performance. Geon‐Hyoung An [22] engineered a mesoporous structured AC (MSAC) through the dehydrogenation process of polyvinylpyrrolidone (PVP) coated AC. PVP aggregates on the surface of commercial AC were dehydrogenated, resulting in carbon consumption and the formation of mesopores within the microporous AC structure (Figure 3c). The optimized structure (MSAC‐1) contained an even fraction of micro‐ and mesopore volumes compared with a micropore‐dominant, unactivated commercial AC, while also incorporating extensive oxygen doping, which enhanced the wettability of the electrode (Figure 3d). The MSAC‐1 cathode demonstrated excellent performance (176 mA h g^−1^ at 0.5 A g^−1^), outperforming commercial AC (125 mA h g^−1^ at 0.5 A g^−1^), due to the combined improvement in ionic diffusion capability from ample mesopores and enhanced ionic transport capability from better hydrophilicity (Figure 3e). It should be noted that the addition of macropores (pore size > 50 nm) has also been reported to improve electrode performance by serving as electrolyte reservoirs throughout the carbon material and facilitating electrolyte percolation [83]. However, their direct impact appears less obvious, given their low SSA and limited contribution to capacitance; hence, the limited reports on macroporous carbon materials for use in ZHSCs.

Zhou et al. [23] synthesized a hierarchical porous AC (HPAC) through an in‐situ two‐step activation procedure during the carbonization of asphalt with potassium hydroxide (KOH). By incorporating a pre‐carbonization step at a lower temperature (400°C), the carbonized asphalt could stabilize and control the size distribution of the nanoporous structure before being fully carbonized and activated by KOH at a higher temperature (900°C). Scanning electron microscopy (SEM) images display HPAC with a textured surface (Figure 3f). The resultant structure exhibited an ultrahigh SSA of 3525 m^2^ g^−1^, comprising a significant amount of both micro‐ and mesopores (Figure 3g), and thus providing abundant active sites and enabling fast kinetics. As a result, HPAC was used as the cathode material in ZHSC, paired with a zinc metal anode and a 3 m zinc triflate (Zn(CF_3_SO_3_)_2_) electrolyte. The cathode exhibited excellent specific capacities of up to 231 mA h g^−1^ at a current density of 0.5 A g^−1^, which was superior to that of a micropore‐dominant commercial AC cathode (76.5 mA h g^−1^). Additionally, the HPAC cathode could retain impressive capacities of 119.4 mA h g^−1^ at an ultrahigh current density of 20 A g^−1^ (Figure 3h), as well as hold ultra‐long cycling stability with a capacity retention of ≈70% after 18 000 cycles at 10 A g^−1^. Similarly, Wang et al. [24] reported various porous carbons (MPCs) using tetra‐alkali metal pyromellitic acid salts as precursors (Figure 3i). The optimized rubidium‐activated carbon (RbPC) was fabricated via the carbonization and self‐activation of a tetra‐alkali rubidium pyromellitic acid salt precursor, resulting in a highly disordered, amorphous carbon structure with interconnected macro‐ and mesopores in the bulk, as well as well‐developed microporosity. Differential functional theory (DFT) calculations on the N_2_ adsorption/desorption measurements revealed pore diameters centered at 0.8, 1.3, and 4.2 nm in RbPC (Figure 3j), indicating its hierarchical porosity, while X‐ray photoelectron spectroscopy (XPS) measurements highlighted good oxygen doping, which would benefit the ionic conductivity and accessibility of RbPC. As the active cathode material, the assembled ZHSCs delivered an enhanced capacity of 247.6 mA h g^−1^ at a current density of 1 A g^−1^. Even at very high current densities, RbPC‐based ZHSCs could maintain capacities of 130.6, 110.8, and 72.2 mA h g^−1^ at 60, 70, and 120 A g^−1^, respectively. Moreover, the RbPC‐based ZHSCs demonstrated impressive performance at high mass loadings (Figure 3k), further suggesting that the hierarchical porosity and abundant oxygen functional groups of RbPC boost its charge transfer kinetics.

Summarily, ACs are porous carbon materials, made from a simple and inexpensive carbonization‐activation method, that can be effectively used as cathode materials in ZHSCs. Initial successful applications of AC cathodes marked a breakthrough for ZHSCs, indicating their viability for practical applications and generating interest in the field. By adjusting the reaction parameters for the synthesis of ACs, modulation over pore sizes and structures can be achieved, leading to considerable enhancements in performance. Notably, the hierarchical pore structure, featuring a combination of micro‐ and mesopores, was found to enhance specific capacity and ionic conductivity, thereby increasing energy and power densities, respectively. In addition, the inclusion of macropores (pore size > 50 nm) can serve as electrolyte reservoirs throughout the carbon material. However, while ACs offer the advantages of abundant pores and low cost, their unstructured pore formation process through carbon etching involves complicated chemical reactions at different temperatures, making the construction of pores difficult to control and predict precisely. This can lead to broad pore size distributions and ambiguous pore structures, hindering its ability for detailed tunability. Additionally, common chemical activators such as KOH are toxic and corrosive, while the harsh consumption of carbon can lead to low yields of the final product (< 10%), which are significant drawbacks in a practical sense. Regardless, ACs remain the primary contender for ZHSC cathodes, exhibiting high capacities with simple production and potential for scale‐up. A vast number of different ACs exist due to diverse raw materials, morphologies, and fabrication processes. Continued application, optimization, and studies of various existing and novel ACs as cathode materials look promising in discovering high‐performing ZHSCs.

### 2.2 Template‐Assisted Porous Carbon

Unlike the carbonization‐activation method used for ACs, which relies on the uncontrolled removal of carbon via physical or chemical means, various template methods have been utilized as flexible strategies for the fabrication of porous carbon materials with narrow pore‐size distributions and well‐defined pore structures [84]. In this approach, a carbon precursor is introduced into or assembled around a template, then carbonized, and the template is removed, leaving behind porous volumes and structures that replicate the template's shape. By utilizing a template as a sacrificial rigid scaffold to create and preserve pore architectures, precise control over pore sizes can be achieved through modification of the template used, such as its dimensions and assembly behavior, enabling the fabrication of carbons with narrow pore‐size distributions and tunable microstructures. Additionally, the directed synthesis of unique morphologies, such as hollow spheres, 2D sheets, and 3D interconnected networks, can be designed to enhance ionic and electrical conductivities by reducing ion diffusion distances and providing continuous electron‐transport pathways. Rational design and assembly of template‐assisted porous carbon materials have been utilized as effective cathode materials for ZHSCs.

#### 2.2.1 Hard Template

The use of hard templates for preparing porous carbons has been regularly explored. The hard template method involves using a desirable solid template in which carbon precursors are impregnated/coated and subsequently carbonized. After carbonization, the solid template is then removed, typically via etching with an acid or alkali solution, leaving behind the template‐assisted carbon material.

**FIGURE 4:**
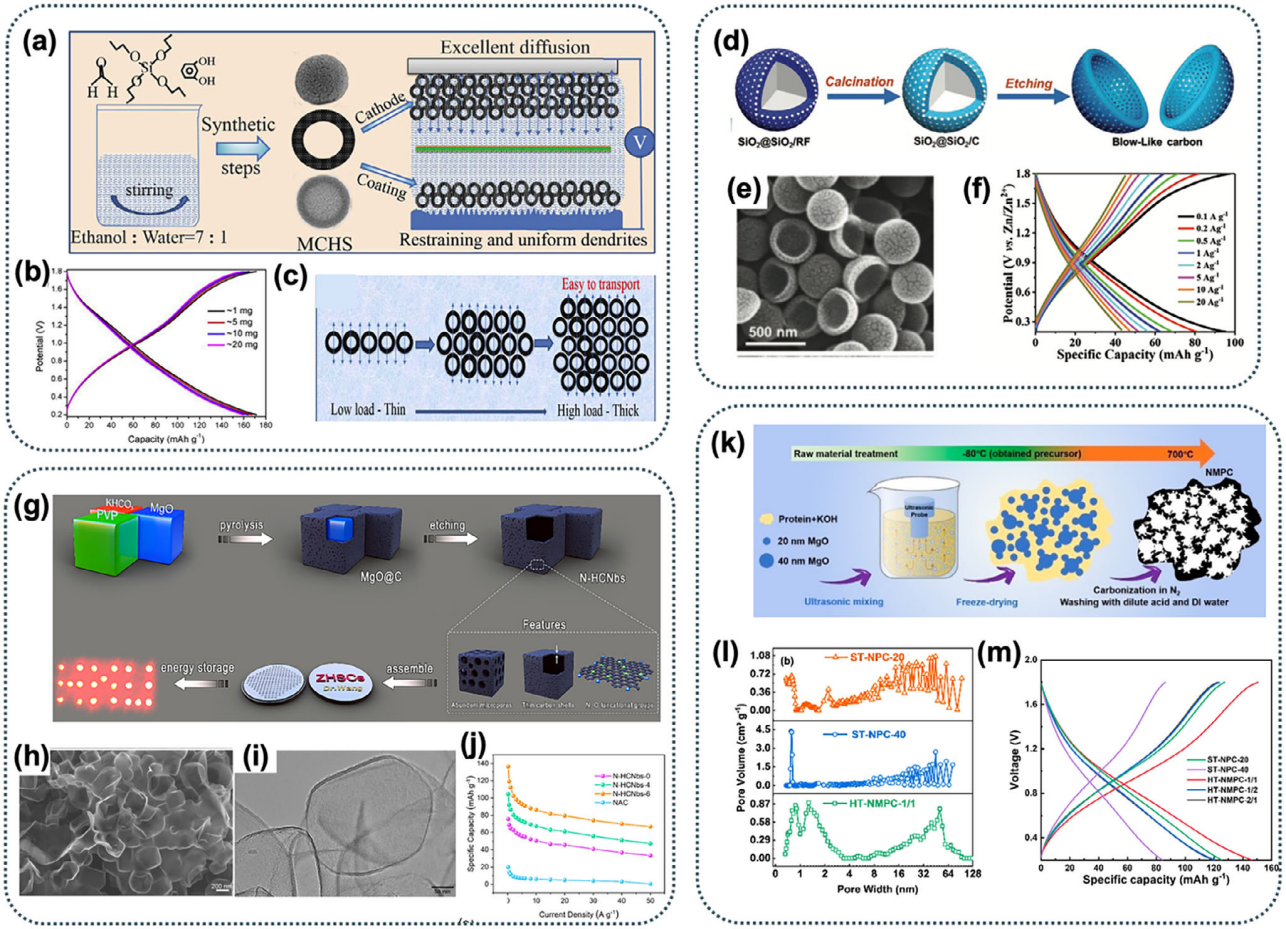
(a) Fabrication process and application of MCHSs. (b) GCD curves of MCHS‐based ZHSC at different mass loading (current density of 0.1 A g^−1^). (c) Scheme of the ion transport process in MCHSs at different mass loadings. Reproduced with permission [25]. Copyright 2020, Elsevier. (d) Formation process of HBCs. (e) SEM image of HBCs. (f) GCD curves of HBC‐based ZHSCs at different current densities. Reproduced with permission [26]. Copyright 2020, Wiley‐VCH. (g) Fabrication process and application of N‐HCNbs. (h) FESEM image and (i) TEM image of N‐HCNbs‐6. (j) Specific capacities of N‐HCNbs‐based ZHSCs at a range of current densities (0.2 – 50 A g^−1^). Reproduced with permission [28]. Copyright 2021, Wiley‐VCH. (k) Fabrication process of HT‐NMPC‐1/1. (l) Calculated PSD of ST‐NPC‐20, ST‐NPC‐40, and HT‐NMPC‐1/1. (m) GCD curves of ZHSCs. Reproduced with permission [30]. Copyright 2025, Wiley‐VCH.

Liu et al. [25] fabricated mesoporous carbon hollow spheres (MCHSs) using silica (SiO_2_) as the hard template (Figure 4a). Spherical SiO_2_ cores were co‐coated with resorcinol/formaldehyde (RF) resins and SiO_2_ pore‐forming particles, forming SiO_2_@SiO_2_/RF core‐shell structures. Subsequent carbonization and removal of the SiO_2_ template presented MCHSs with a large SSA of 1275 m^2^ g^−1^, pore volume of 1.73 cm^3^ g^−1^, and a hierarchical pore structure with pore sizes centered around 1.2, 4.6, and 10.7 nm. When used as the active cathode material in ZHSCs, excellent performance was demonstrated, benefiting from the MCHS's high surface area and abundant pores. The ZHSC exhibited a high specific capacity of 174.7 mA h g^−1^ at a current density of 0.1 A g^−1^ with excellent cycling stability, retaining 100% capacity over 10 000 cycles at 1 A g^−1^. Moreover, the cathode performance is maintained at high mass loadings (from 1 to 20 mg cm^−2^), with the highly interconnected hollow structure of MCHSs allowing rapid ion transport and mitigating diffusion limitations throughout the entire electrode framework (Figure 4b,c). Similar utilizations of silica as the hard template for synthesizing hollow carbon structures have been demonstrated and applied. For instance, Fei et al. [26] also utilized SiO_2_@SiO_2_/RF core‐shell structures to synthesize hollow bowl‐like carbon (HBC) (Figure 4d). HBCs demonstrated good performance as cathode materials in ZSHC (Figure 4f), with their hollow porous shape (Figure 4e) providing strong structural stability, high electrical conductivity, large SSA, and enabling an ion buffer to promote rapid mass ion migration. More recently, Du et al. [27] employed a SiO_2_ hard template to synthesize double‐shell nitrogen‐doped hollow carbon spheres (HSCs). By confining spherical PDA precursors with a coating of mesoporous silica shell, followed by pyrolysis and silica etching, HSCs with a double shell morphology were obtained, containing a microporous/mesoporous shell structure. As the cathode material in ZHSCs, optimized HSC_t6_ delivered high capacitances up to 260 F g^−1^ at a current density of 0.1 A g^−1^ and excellent cycling stability, achieving 99.3% capacity retention after 30 000 cycles at 2 A g^−1^.

Due to the size restrictions of synthesizing templates, meso‐ and macropores are typically the targets for formation within these methods. Therefore, the inclusion of an activator is common for the development of micropores. Chen et al. [28] utilized magnesium oxide (MgO) as the hard template for the synthesis of nitrogen‐doped hollow carbon nanoboxes (N‐HCNbs) (Figure 4g). A mixture containing PVP, MgO, and potassium bicarbonate (KHCO_3_) was used, serving as the nitrogen‐containing carbon source, the hard template, and the activator, respectively. When heated, the PVP could melt onto the MgO template and carbonize, while KHCO_3_ could decompose and develop micropores within the carbonised structure. The resultant N‐HCNbs, after removal of the MgO template, exhibit interconnected hollow structures (Figure 4h,i) composed of ultra‐thin‐walled nanoboxes with thicknesses of 8–10 nm. The interconnected hollow structure could facilitate fast electron transfer between nanoboxes, while ultra‐thin walls can shorten the diffusion length of ions, endowing N‐HCNbs with fast electrochemical kinetic characteristics. The optimized porous carbon, N‐HCNbs‐6, exhibited a high SSA of 1660.2 m^2^ g^−1^ and moderate amounts of nitrogen (5.2%) and oxygen (10.9%) doping, allowing for abundant active sites and the contribution of pseudocapacitance, respectively. As a result, the assembled ZHSC with N‐HCNbs‐6 cathode exhibited high specific capacities of up to 136.2 mA h g^−1^ at a current density of 0.2 A g^−1^, as well as excellent rate capabilities, retaining 66.7 mA h g^−1^ at 50 A g^−1^ (Figure 4j). Likewise, 3D honeycomb‐like hierarchically porous carbons (HHPCs) were synthesized using a similar mixture of MgO template, KHCO_3_ activator, and glucose as the carbon source [29]. After pyrolysis and removal of the template, the optimized HHPCs displayed a large SSA and a continuous interconnected hierarchical structure, achieving a high capacity of 147 mA h g^−1^ at a current density of 0.2 A g^−1^. Gao et al. [30] fabricated nitrogen‐doped multilevel porous carbon (HT‐NMPC‐1/1) using MgO particles as hard mesoporous templates (Figure 4k). By employing two different‐sized MgO particles (20 and 40 nm) and KOH as the micropore‐forming activating agent, HT‐NMPC‐1/1 could attain a regulated hierarchical pore structure, enhancing ion transfer efficiency and improving the utilization of micropores. Pore size distribution analysis revealed HT‐NMPC‐1/1 had various pore sizes centered around 0.85, 1.27, and 50.39 nm, while single particle size templated‐carbons (ST‐NPC‐20, ST‐NPC‐40) showed limited pore sizes (Figure 4l). The HT‐NMPC‐1/1 delivered impressive performances, achieving a high specific capacity of 146.65 mA h^−1^ g^−1^ at a current density of 0.2 A g^−1^ (Figure 4m), and long‐term cycling stability (93.09% capacity retention after 8000 cycles at 2 A g^−1^), outperforming ST‐NPCs. Meanwhile, Zhang et al. [31] synthesized a self‐designed nitrogen‐doped hierarchical porous carbon (HNPC) using NaY zeolite as a microporous hard template, and furfuryl alcohol (FA) as the carbon source. Post‐ammonia treatment activation was applied to the carbonized materials, resulting in the development of mesopores and nitrogen doping, as evident by pore size analysis and XPS. The ZHSC assembled with HNPC cathode delivered outstanding performance, with capacities reaching 177.8 mA h g^−1^ at a high current density of 4.2 A g^−1^ and demonstrating excellent cycling stability (99.7% capacity retention after 20 000 cycles at 16.7 A g^−1^). Furthermore, the viability of the material for practical applications was demonstrated in a quasi‐solid‐state ZHSC, where the HNPC cathode was paired with galvanized Zn nanosheets as the anode in a PVA gel electrolyte. The quasi‐solid‐state Zn HNPC cell showed impressive energy and power densities (91.8 W h kg^−1^, 27.6 kW kg^−1^), surpassing various other reported EES devices.

**FIGURE 5:**
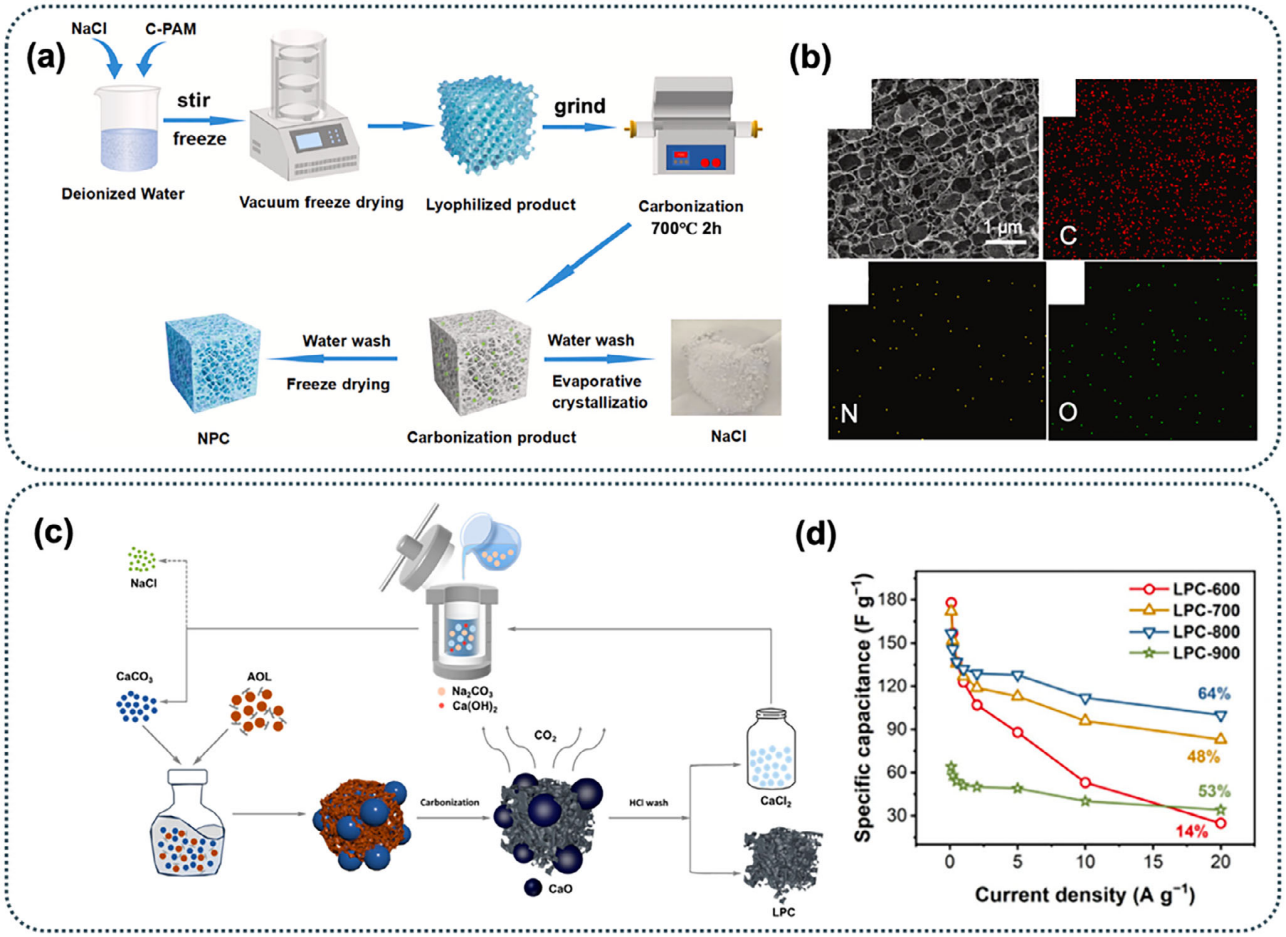
(a) Fabrication and recycling process of NPC and NaCl template, respectively. (b) Elemental mapping images of NPC‐5. Reproduced with permission [32]. Copyright 2024, Elsevier. (c) Fabrication and recycling process of LPC and CaCO_3_ template, respectively. (d) Rate performance of LPC‐based ZHSCs. Reproduced with permission [33]. Copyright 2024, Royal Society of Chemistry.

A significant disadvantage of many hard templates is the loss of template content after use, which increases costs and material waste. Alternatively, recyclable hard templates could solve this issue, making the synthesis of hard template‐assisted porous carbons more sustainable. For example, Song et al. [32] utilized a sodium chloride (NaCl) salt template for the preparation of nitrogen and oxygen co‐doped porous carbon (NPC). Through freeze‐drying a gel mixture of polyacrylamide and NaCl solution, NaCl nanocrystals can form, embedding themselves within the carbon precursor and acting as hard templates. Once carbonized, a simple water wash can dissolve the NaCl template from the NPC, enabling it to be reused (Figure 5a). The optimized NPC‐5 could obtain a well‐developed interconnected porous structure with ample doping (nitrogen 11.38%, oxygen 8.71%), beneficial for its electrochemical performance (Figure 5b). A high energy density of 85.3 W h kg^−1^ was demonstrated when applied as the cathode material in a ZHSC. Meanwhile, Calcium carbonate (CaCO_3_) was used as a recyclable hard template to synthesize lignin‐derived porous carbons (LPCs) by Zhu et al. [33] (Figure 5c). Not only can CaCO_3_ nanoparticles (50 nm) act as pore‐forming templates, but CaCO_3_ can also decompose into CaO, leading to the release of CO_2_ and causing additional activation during carbonization. The CaCO_3_ templates could be recovered after acid pickling through a double decomposition reaction between leftover CaCl_2_ and Na_2_CO_3_. Through this green synthesis strategy, the optimized LPC (LPC‐800) exhibited a significant SSA of 860.5 m^2^ g^−1^, with a predominance of mesopores (90% of the total pore volume). The ZHSC with LPC‐800 as the active cathode material exhibited impressive rate performance, retaining 64% of its original capacity over a current density range of 0.1 to 20 A g^−1^ (Figure 5d). The hierarchical mesoporous structure of LPC‐800 enables rapid and efficient ionic transfer kinetics within the electrode.

By employing the hard template method, facile synthesis of effective carbon cathode materials for ZHSCs can be developed with uniform and controllable pore sizes. However, several challenges exist, particularly associated with hard template synthesis, such as high costs, complex procedures, and time‐consuming steps. These factors hinder its potential for scale‐up and practical application. The removal of hard templates also presents difficulties, often involving the use of hazardous etching agents, such as hydrogen fluoride (HF), and risking damage to fragile carbon structures. Despite these disadvantages, the hard template method offers a versatile approach for creating diverse carbon pore structures, which can be utilized in ZHSC research. Further development and use of novel hard templates could aid in gaining a deeper understanding of the relationship between pore structures and cathode performance.

#### 2.2.2 Soft Template

In contrast to hard templating, soft templating of carbon materials offers an alternative approach to synthesizing controlled pore structures while avoiding the use of harsh chemicals typically needed for hard template removal [85]. In the soft template method, templates of organic molecules or supramolecules with functional groups are commonly used. When paired with a suitable solvent, these functional groups can generate strong intermolecular forces, such as hydrogen bonding, hydrophilic/hydrophobic interactions, or electrostatic interactions, allowing for the formation of micelle‐like aggregates that serve as templates for carbon precursors. The subsequent carbonization process decomposes or evaporates the soft templates, eliminating the need for a separate template removal step as required in the hard template method, and produces ordered porous carbon materials with distinctive architectures.

**FIGURE 6:**
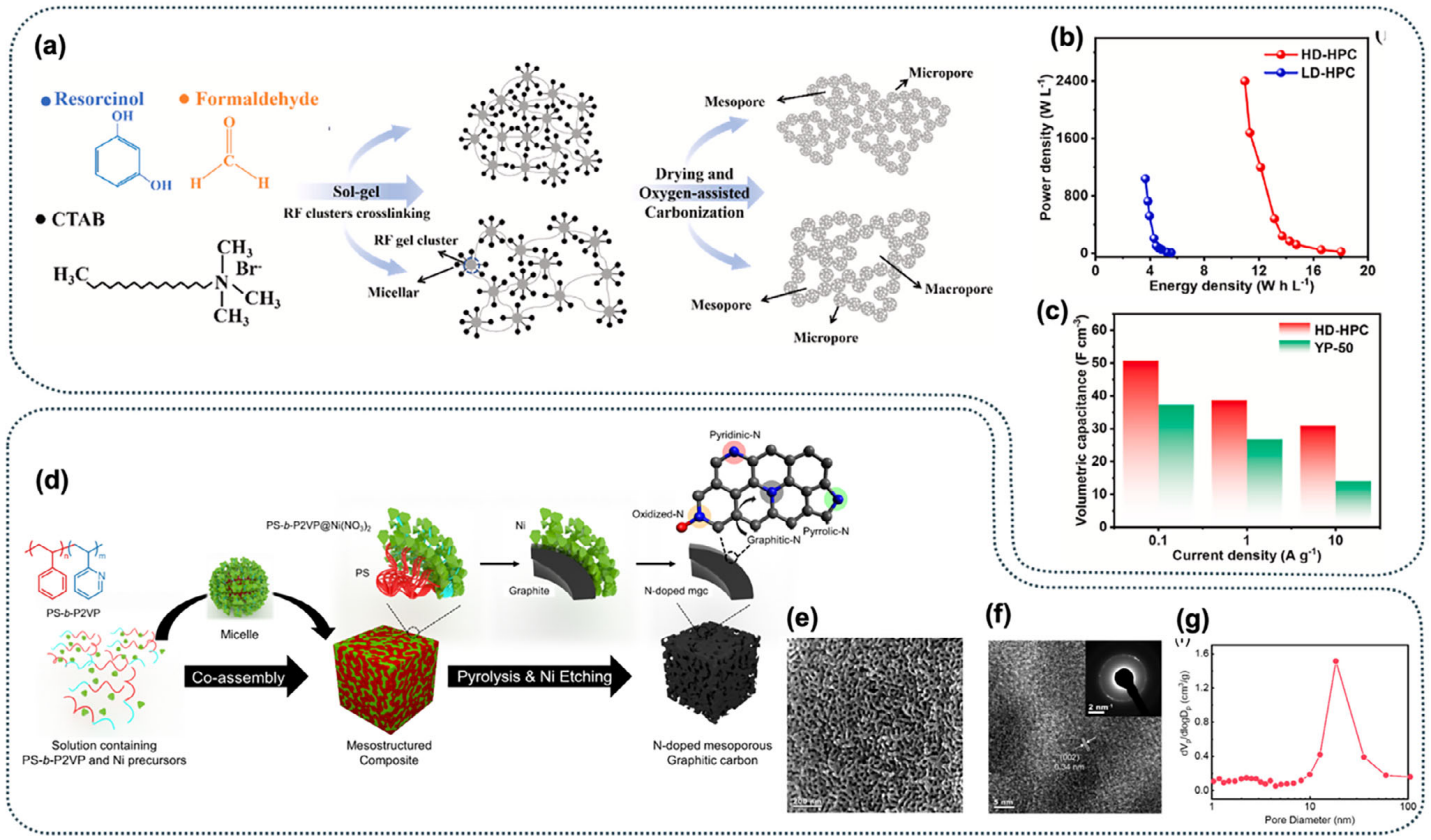
(a) Fabrication process of HPCs with differing densities. Performance of HD‐HPC‐based ZHSCs compared with (b) LD‐HPC and (c) YP50. Reproduced with permission [34]. Copyright 2025, Elsevier. (d) Fabrication process of N‐mgcs. (e) SEM image and (f) HRTEM image with inset of SAED pattern of N‐mgc. (g) Calculated PSD of N‐mgc. Reproduced with permission [35]. Copyright 2023, American Chemical Society.

Recently, Li et al. [34] synthesized hierarchical porous carbons (HPCs) using a surfactant‐mediated crosslinking strategy during the phenolic resin sol‐gel process (Figure 6a). The surfactant, cetyltrimethylammonium bromide (CTAB), was employed to form micelles, acting as a soft template for dispersing RF gel clusters and aggregates during sol‐gel polymerization, leading to the formation of a three‐dimensional nano‐network with interconnected interstitial pores. After oxygen‐assisted carbonization to create micropores, HPCs were formed. The pore size distributions can be easily tuned by altering the molar ratio of CTAB to R. Specifically, the mesopore size can be decreased by increasing the CTAB/R ratio, resulting in denser materials. The denser HPC (HD‐HPC) based ZHSC achieved a good volumetric energy density of 18 W h L^−1^ at a volumetric power density of 24 W L^−1^, outperforming less dense HPC (LD‐HPC) as well as a commercial microporous carbon (YP50) (Figure 6b,c). Kim et al. [35] utilized polystyrene‐*block*‐poly(2‐vinylpyridine) (PS‐b‐P2VP) block copolymers to prepare nitrogen‐doped mesoporous graphitic carbon (N‐mgc) (Figure 6d). The PS‐b‐P2VP block copolymer co‐assembled with nickel nitrate to form a mesostructured composite, where the amphiphilic block copolymer acted as both soft template and carbon/nitrogen source, and nickel acted as the catalyst for graphitization during carbonization. The resultant N‐mgcs contained an interconnected mesoporous structure with a uniform pore size of 18 nm (Figure 6e–g). When applied as the cathode material in ZHSCs, the ZHSC showed good energy storage performance, with an energy density of 19.4 W h kg^−1^ at a power density of 180 W kg^−1^. Meanwhile, C_3_N_4_ was employed as a soft template for the synthesis of nitrogen‐doped porous carbon (NPC) cathode by Cao et al. [36] C_3_N_4_ not only functions as a soft template but also serves as a nitrogen source, as it decomposes during carbonization. Mixed with glucose and KOH, as the carbon source and activator, respectively, the optimized NPC‐2 showed a significant SSA of 2407 m^2^ g^−1^ and high nitrogen doping of 10.1%. The large surface area and elevated nitrogen content contributed to improved electrochemical performance, achieving a maximum specific capacity of 166.5 mA h g^−1^ when assembled in a ZHSC.

**FIGURE 7:**
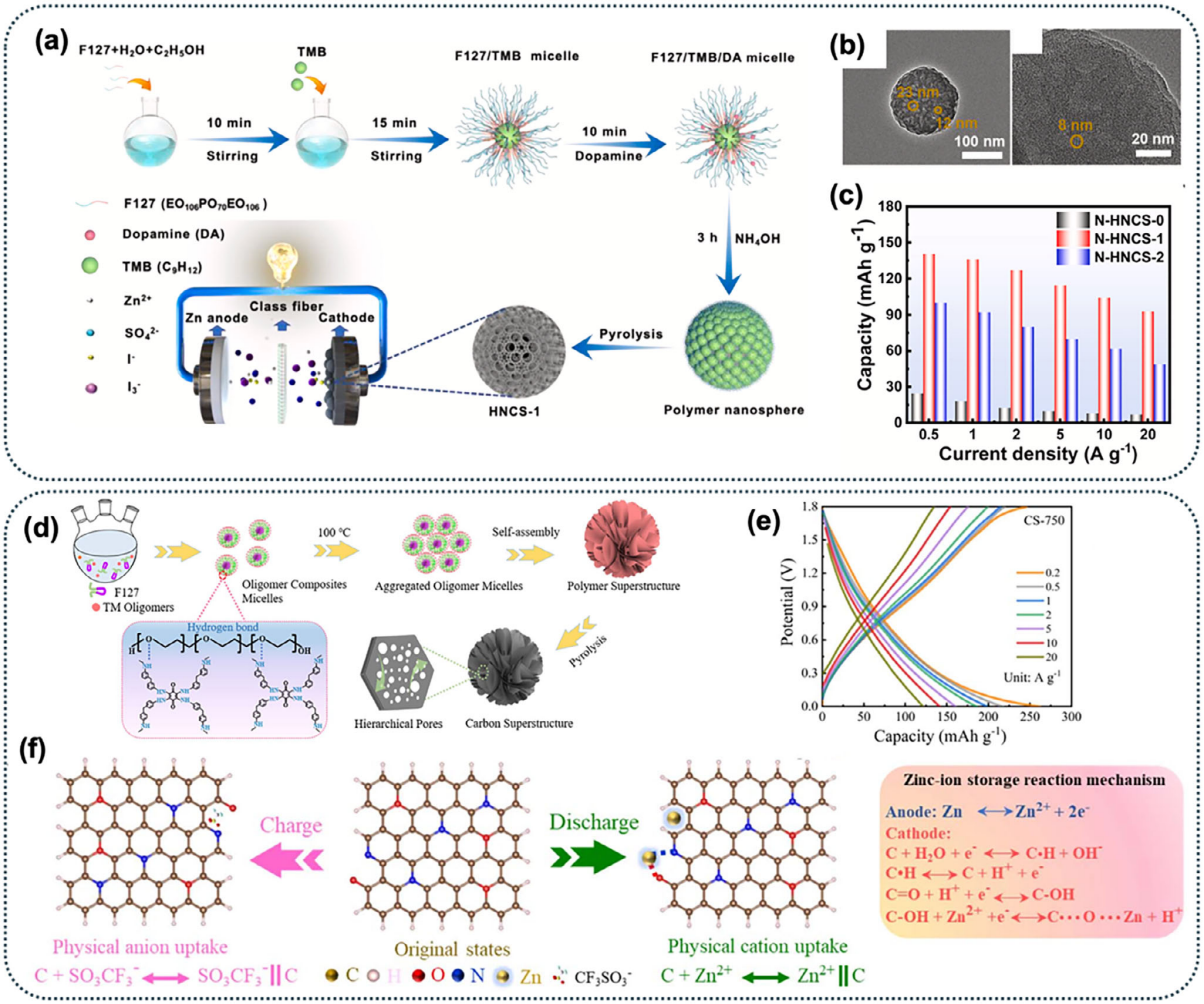
(a) Fabrication process and application of N‐HNCS‐1. (b) TEM images of N‐HNCS‐1. (c) Rate performances of N‐HNCS‐based ZHSCs. Reproduced with permission [37]. Copyright 2024, Elsevier. (d) Fabrication process of CS‐750. (e) GCD curves of CS‐750‐based ZHSCs at different current densities. (f) Charge storage mechanism of CS‐750 cathode in ZHSC. Reproduced with permission [38]. Copyright 2025, Royal Society of Chemistry.

The use of Pluronic F127 triblock copolymer has been regularly demonstrated as an effective soft template for the synthesis of porous carbons. Yang et al. [37] prepared N‐doped porous carbon spheres (N‐HNCSs) using Pluronic F127 as the structure‐guiding agent (soft‐ template), 1,3,5‐trimethylbenzene (TMB) as the pore‐expanding agent, and polydopamine (DA) as the carbon/nitrogen source (Figure 7a). Assembled F127/TMB/DA micelles could form polymer nanospherical superstructures, which were carbonized to produce N‐HNCSs with adjustable structures and sizes, depending on the amount of TMB used. N‐HNCS‐1, synthesized with moderate TMB, exhibited a golf‐ball‐like morphology with a hierarchical porous structure (Figure 7b), and pore sizes ranging from 2 to 50 nm. Assembled ZHSCs with an N‐HNCS‐1 cathode delivered impressive performance, demonstrating a specific capacity of 142 mA h g^−1^ at a 0.5 A g^−1^ current density. Even when the current increased to 20 A g^−1^, the cell maintained a high capacity of 94 mA h g^−1^ (Figure 7c). Recently, Jha et al. [38] utilized F127 triblock copolymer and tetrachlorobenzoquinone–4,4′‐methylenedianiline (TM) oligomers to synthesize flower‐like hierarchical porous carbon superstructures (CS‐750) (Figure 7d). Oligomer composite micelles could form and aggregate, assembling into a flower‐shaped structure, whereupon carbonization, CS‐750 was synthesized with interconnected structures and a high SSA of 2824 m^2^ g^−1^. The CS‐750‐based ZHSC delivered a remarkable energy density of 160.8 W h kg^−1^, with a high discharge capacity of 262.8 mA h g^−1^ (Figure 7e). Excellent cycling stability was also demonstrated, achieving 93.2% capacity retention after 200 000 cycles at 20 A g^−1^. The robust carbon superstructure of CS‐750, characterized by highly interconnected channels and abundant active sites, facilitates rapid ion migration and superior charge‐storage capability, thereby ensuring a large energy density. Further mechanistic studies revealed that the additional adsorption of H^+^ ions significantly contributed to enhancing the capacity and rate performance, alongside the primary adsorption of zinc ions/anions (CF_3_SO_3_^−^), highlighting the crucial role of the proton‐assisted zinc‐ion‐storage process (Figure 7f).

The soft template method allows facile preparation of hierarchical porous carbon materials with interesting structures. The soft templates used can readily decompose, usually through pyrolysis, making the process more sensitive and environmentally friendly compared to traditional hard templates. Additionally, pore sizes can be easily modified by altering the mixing ratios (e.g., solvent and micelles), temperatures, and solvents in the synthesis procedure. However, developing soft templates is complex and stringent, requiring strict adherence to specific conditions for success. Specifically, the template and carbon precursor must interact and self‐assemble into nanostructures with components that can form pores, and they must have complementary thermal stabilities, with the carbon precursor able to withstand template removal without collapsing. Due to these strict requirements, the number of available soft templates is limited. As‐prepared porous carbons are also predominantly mesoporous, with limited surface area and capacity, which restricts their wider exploration in ZHSCs and EES devices. Nonetheless, the soft‐template method offers a more sustainable approach to creating diverse porous carbon materials with tunable structures, and further development and application are warranted.

#### 2.2.3 Other Template

Novel processes have emerged in recent years for synthesizing porous carbons, including the use of unique templates that differ from traditional hard or soft templates and have been employed in the development of cathode materials for ZHSCs. For example, a dynamic dual template method was used in synthesizing porous carbon nanorods (HPCNs) by Gao et al. [39]. Sphere‐shaped mesomorphous complexes, formed by self‐assembly of CTAB and polyacrylic acid (PAA), were further assembled into nanorods under the structure‐directing agent of Pluronic P123, utilizing dual dynamic soft templates. A silica skeleton was added to the polymeric nanorods by the addition of tetraethyl orthosilicate (TEOS), allowing for the infiltration of the carbon precursor (sucrose), and subsequent carbonization and removal of the silica, formed HPCNs. Upon further activation with KOH, activated HPCN cathodes delivered large capacities up to 157 mA h g^−1^ in a ZHSC setup, as well as excellent cycling stability, retaining nearly 100% capacity after 10 000 cycles at 5 A g^−1^. Meanwhile, Xing et al. [40] utilized a bubble‐templated polymerization of pyrrole (py) to carbonize and synthesize an N‐doped carbon nanosheet aerogel (N‐CNAG) cathode for ZHSCs. The N‐CNAG exhibited a 3D honeycomb‐like structure, constructed of stacked carbon nanosheets with defective holes. The N‐CNAG‐based ZHSCs demonstrated outstanding performance, delivering a capacitance of 706 F g^−1^, which is attributed to the high active nitrogen content and hierarchically porous structure of the N‐CNAGs. Ice‐templated porous carbon cages (PCC‐3) were used as cathode materials for ZHSC by Xue et al. [41]. By freezing a mixture of aqueous KOH solution with the carbon precursor (heavy bio‐oil), formed ice crystals could template the precursor. Subsequent freeze‐drying and carbonization/activation resulted in hollow, interconnected, cage‐like porous carbon structures. Assembled ZHSCs with PCC‐3 achieved a high energy density of 119 W h kg^−1^ at a power density of 355.17 W kg^−1^.

In summary, the template method is a versatile approach for preparing porous carbons with narrow pore size distributions and unique morphologies. It is particularly effective at creating mesoporous structures with good tunability, and when combined with methods for micropore formation, such as chemical activation, carbons with the desirable hierarchical porous structure can be easily synthesized for use in ZHSCs. Furthermore, the ability to engineer specific carbon morphologies, such as hollow architectures and 3D interconnected frameworks, can enhance electrochemical performance by modulating ion diffusion pathways and electron transport. Although a universal consensus on the optimal structural design has not yet been reached, hierarchical porous structures with interconnected networks are frequently reported to be effective for balancing capacity and rate performance. Nevertheless, designing and synthesizing templates remains challenging, as many developed templates require time‐consuming processes, high costs, and unsustainable procedures, limiting their practical application. Despite these limitations, the ability to produce diverse morphologies with controllable structures can offer a wide variety of porous carbons for testing, facilitating the discovery and optimization of effective cathode materials for advanced ZHSCs.

### 2.3 Metal‐Organic Framework‐Derived Carbons

Differing from traditional template‐assisted carbons, metal‐organic framework (MOF)‐derived carbons are a type of self‐templated carbon that can exhibit high porosity, large SSA, and well‐defined channels inherited from their MOF precursors, without the need for external templates. MOFs are a type of hybrid material composed of metal nodes or clusters connected by coordination bonds with organic ligands, forming highly ordered and stable open frameworks. The diverse and tunable structures of MOFs enable precise control over the physicochemical properties of the carbonized material, making MOF‐derived porous carbons an ideal choice for SCs and ZHSCs.

**FIGURE 8:**
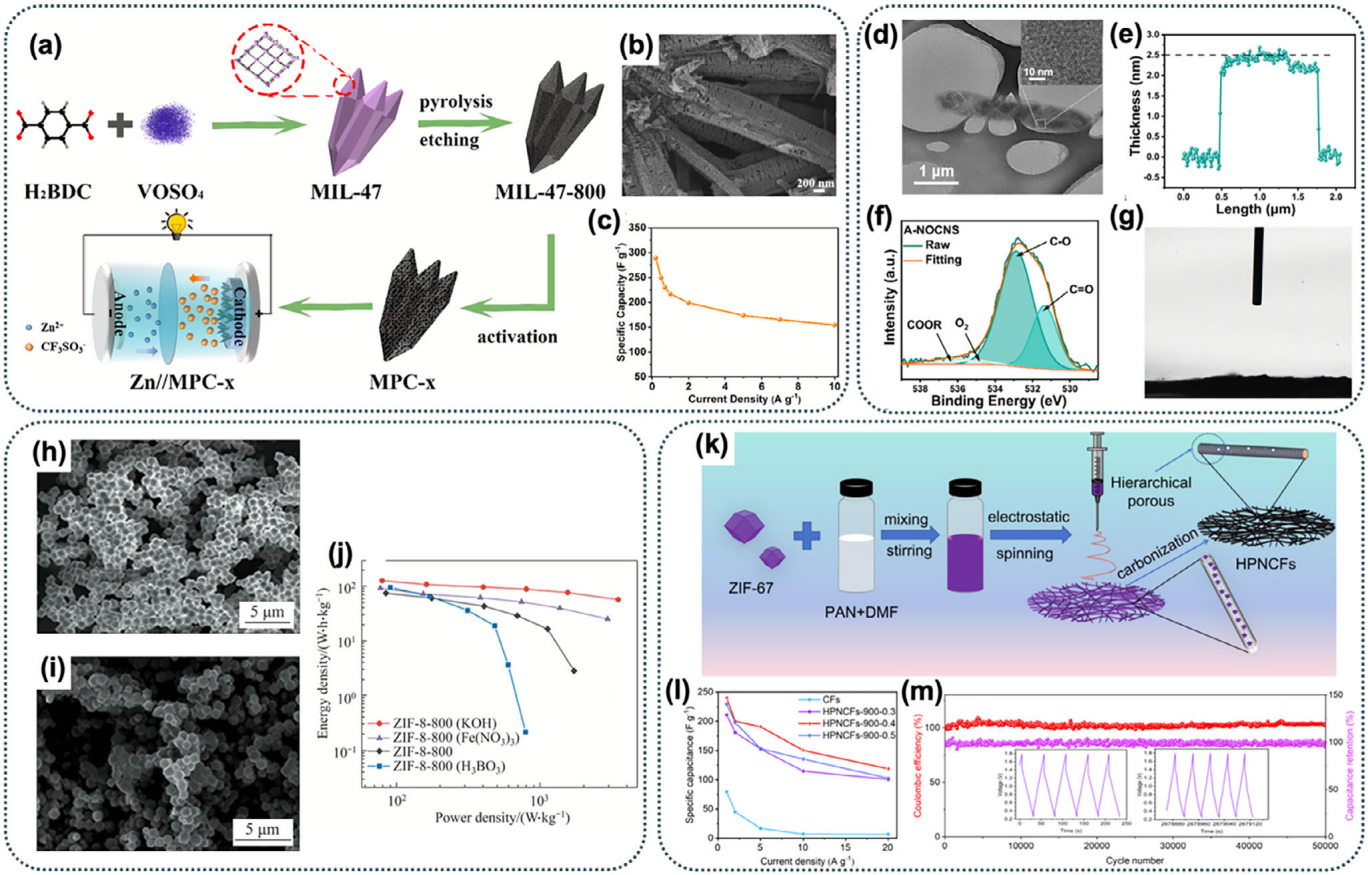
(a) Fabrication process and application of MPCs. (b) SEM image of MPC‐2. (c) Rate capability of MPC‐2‐based ZHSCs. Reproduced with permission [42]. Copyright 2022, Elsevier. (d) TEM and HRTEM (inset) images of A‐NOCNSs. (e) AFM height profile of A‐NOCNSs. (f) High‐resolution O1s XPS spectra of A‐NOCNS. (g) Water contact angle of A‐NOCNS. Reproduced with permission [43]. Copyright 2024, Royal Society of Chemistry. SEM images of (h) ZIF‐8 precursor and (i) ZIF‐8‐800(KOH). (j) Ragone diagram of ZIF‐8‐derived carbon‐based ZHSCs. Reproduced with permission [44]. Copyright 2024, Springer Nature. (k) Fabrication process of HPNCFs. (l) Rate performance of HPNCF‐based ZHSCs. (m) Cycling stability and coulombic efficiency of HPNCFs‐900‐0.4 at 10 A g^−1^. Reproduced with permission [46]. Copyright 2025, American Chemical Society.

For example, a MIL‐47 MOF was carbonized to create a new type of sharpened pencil‐like nanoporous carbon (MPC) (Figure 8a) [42]. The rod‐like morphology with stacked nanosheets was preserved during the carbonization of the original MOF precursor (Figure 8b). With further KOH activation, the optimized MPC‐2 exhibited a large surface area of 2125 m^2^ g^−1^ with a high pore volume of 2.21 cm^3^ g^−1^, offering abundant active sites. As the active cathode material in ZHSCs, the MPC‐2 delivered a high specific capacitance of 289.2 F g^−1^ at a current density of 0.2 A g^−1^, and retained 154.4 F g^−1^ after the current density was increased 50‐fold to 10 A g^−1^ (Figure 8c). Additionally, a 2D‐MOF was utilized as a precursor for preparing oxygen‐rich nitrogen‐doped porous carbon nanosheets (A‐NOCNSs) by Han et al. [43] 2D structures, compared to 3D ones, can have a higher accessible surface area with fully exposed surface active sites for efficient ion diffusion and pseudocapacitance, beneficial for enhancing electrochemical performance. The 2D nanosheet micromorphology was maintained after carbonization of the Zn(bim)(OAc) MOF, and after KOH activation, A‐NOCNSs displayed an ultra‐thin thickness of 2.5 nm with a hierarchical pore structure (Figure 8d,e). In addition, XPS analysis revealed a rich oxygen content of 33.39 at%, endowing A‐NOCNSs with super‐hydrophilicity, thereby boosting ion migration rates and improving reaction kinetics (Figure 8f,g). Consequently, A‐NOCNS‐based ZHSCs achieved a maximum energy density of 162.88 W h kg^−1^, an excellent power density of 28.43 kW kg^−1^, and impressive long‐term cycling stability (90.23% capacity retention after 20 000 cycles at 10 A g^−1^).

Due to their porous structures and high nitrogen content, zeolitic imidazolate frameworks (ZIFs) have been regularly explored as a platform for the synthesis of nitrogen‐doped porous carbon materials. Liu et al. [44] utilized ZIF‐8 MOFs as a precursor for the fabrication of nitrogen‐ and oxygen‐doped porous carbons. The prepared MOFs exhibited a well‐defined rhombic dodecahedral shape with a uniform size distribution (Figure 8h). After carbonization and further activation with KOH, nitrogen‐oxygen‐doped porous carbons (ZIF‐8‐800(KOH)) were obtained, maintaining the original ZIF‐8 shape (Figure 8i) and displaying a significant surface area of 2814.67 m^2^ g^−1^ with a rich pore structure. As the active cathode material in ZHSCs, ZIF‐8‐800(KOH) exhibited excellent performance, achieving a high energy density of 125 W h kg^−1^ at a power density of 79 W kg^−1^ (Figure 8j). Wang et al. [45] fabricated zinc/nitrogen co‐doped porous carbons (ZMDPC) through carbonization of in‐situ grown ZIF‐8 MOFs on prepared carbon nanorods. The optimized ZMDPC‐700 displayed a high SSA of 1190.9 m^2^ g^−1^, with pore sizes extending from 1.6 to 7.8 nm, and a high nitrogen doping of 24.61%. A ZHSC consisting of ZMDP‐700 cathode, zinc foil anode, and 2 m ZnSO4 electrolyte delivered large capacities up to 255.6 F g^−1^ at a current density of 0.5 A g^−1^, along with excellent cycling stability, retaining 97.8% capacity after 10 000 cycles at 10 A g^−1^. Recently, Cui et al. prepared a freestanding cathode from entangled 3D networks of hierarchical porous nitrogen‐doped carbon nanofibers (HPNCFs) [46]. The HPNCFs were synthesized through electrostatic spinning and subsequent carbonization of ZIF‐67 nanoparticles embedded in polyacrylonitrile, and displayed textual fibers with a rich hierarchical pore structure (Figure 8k). The fibrous nature enables HPNCFs to be used as a freestanding cathode without needing additives or current collectors, simplifying electrode manufacturing and reducing electrode mass. The HPNCF‐based ZHSC demonstrated an excellent specific capacitance of 240 F g^−1^ at 1 A g^−1^ and showed superior cycling stability, maintaining a capacity of 99.58% after 50,000 cycles with a coulombic efficiency of nearly 100% (Figure 8l,m).

MOF‐derived carbons enable precise tuning of microporous structures and chemical properties through their MOF precursors, which is not commonly achievable with other pore‐forming methods. This distinct advantage makes MOF‐derived carbons attractive for developing advanced carbon cathode materials for ZHSCs. However, the ordered structure in MOFs can produce carbons with limited pore sizes, rendering them unsuitable for efficient ion transport and adsorption. This often requires additional activation to create effective carbon cathodes, thereby losing the initial benefit. Moreover, the costly and complex synthesis of MOFs makes MOF‐derived carbons less attractive for practical applications. Continued development of novel MOF materials with diverse pore sizes and structures is crucial to fully harness their carbonized form.

### 2.4 Biomass‐Derived Carbon

Not only is the pore‐forming method important for the characteristics of porous carbon materials, but the carbon precursors also significantly vary the properties, such as chemical composition, degree of graphitization, and physicochemical stability [86, 87]. As a cost‐effective and renewable carbon source, carbon‐rich biomass precursors have attracted widespread interest from researchers, offering diverse and plentiful raw materials, low costs, and environmental sustainability [88]. Through carbonization with activation and/or templating, biomass‐derived porous carbons can be obtained with high SSA, rich porosity, and tunable structures, presenting as promising eco‐friendly candidate materials for cathodes in ZHSCs.

**FIGURE 9:**
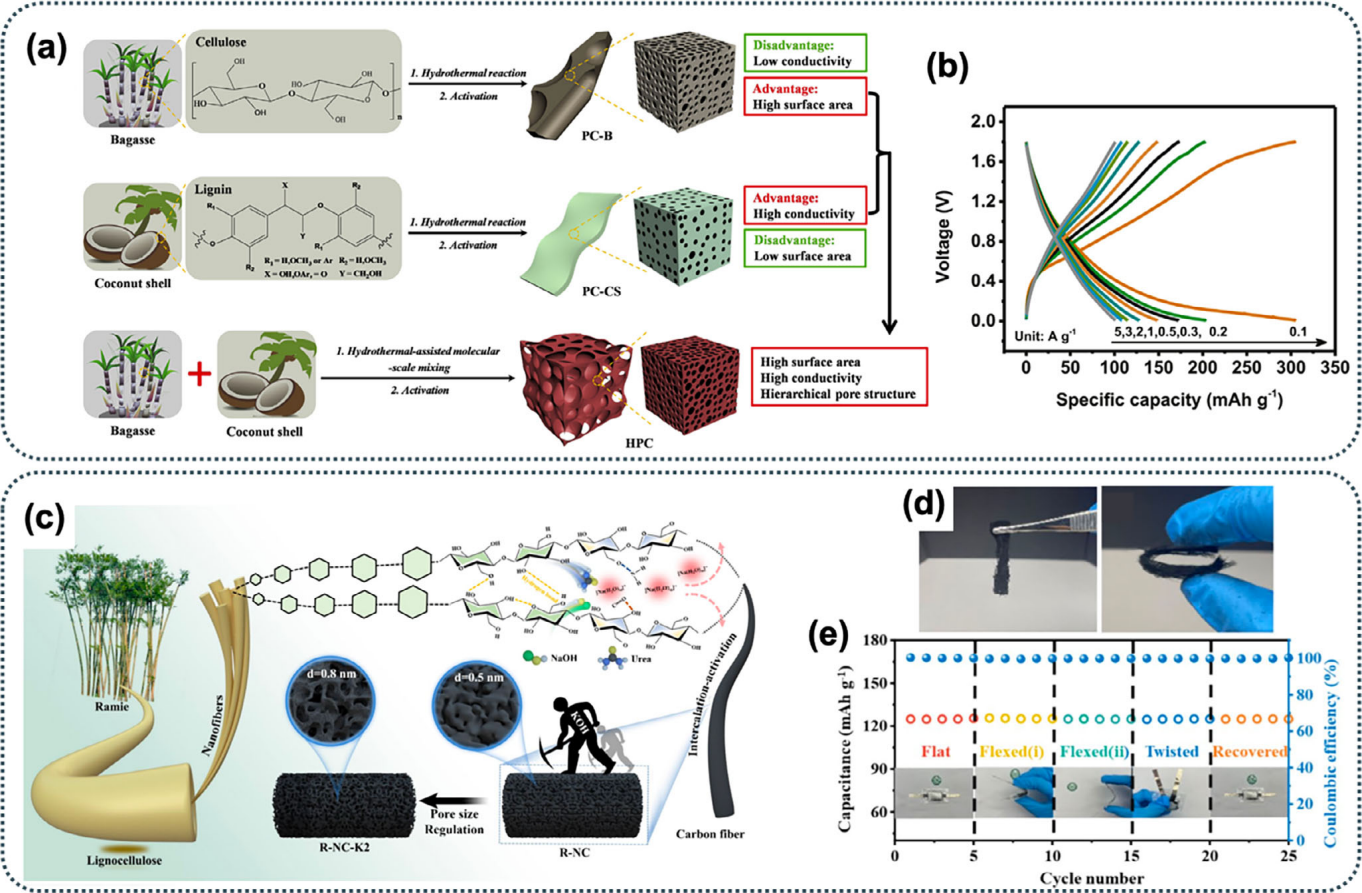
(a) Fabrication process and characteristics of HPC from respective precursors. (b) GCD curves of HPC‐based ZHSC at various current densities. Reproduced with permission [48]. Copyright 2019, Elsevier. (c) Fabrication process of R‐NC‐K2. (d) Photo of freestanding and flexible R‐NC‐K2 cathode. (e) Specific capacity of flexible R‐NC‐K2‐based ZHSC under different bending conditions. Reproduced with permission [49]. Copyright 2025, Springer Nature.

Lignocellulosic biomass, composed primarily of cellulose, lignin, and hemicellulose, is one of the most abundant renewable resources in the world [89]. The natural polymers that make up the biomass can serve as good carbon sources with oxygen dopants to synthesize a diverse range of functional materials. Yang et al. [47] utilized wood sawdust as lignocellulosic biomass precursor for the synthesis of porous carbon (LDC) electrodes. The wood sawdust precursor was hydrothermally treated with a concentrated zinc chloride (ZnCl_2_) solution, and subsequent carbonization yielded LDCs with abundant mesopores and oxygen functional groups. The ZnCl_2_ acted as a molten salt template and an activating agent during carbonization. As a result, high capacitances of up to 263 F g^−1^ were exhibited by LDC cathodes in ZHSCs at a current density of 0.5 A g^−1^, as well as 172 F g^−1^ at 10 A g^−1^. Moreover, Yu et al. [48] achieved high‐energy‐density ZHSCs by employing a hierarchical porous carbon (HPC) derived from a mixture of bagasse and coconut shell biomass precursors as the cathode material (Figure 9a). The researchers found that porous carbons derived from cellulose‐ and hemicellulose‐rich bagasse had a high surface area but low electrical conductivity, while porous carbons derived from lignin‐rich coconut shells had high conductivity but a low surface area. By mixing the two precursors, favorable properties of both high SSA and high conductivity could be attained for HPCs, resulting in HPC‐based ZHSCs with superior specific capacities up to 305 mA h g^−1^ (Figure 9b). More recently, ramie—a typical bast fiber—was used as a lignocellulose biomass precursor for the synthesis of a freestanding, flexible, and porous carbon electrode (Figure 9c,d) [49]. To engineer pores into the dense supramolecular structure of ramie fiber material, a top‐down intercalation activation strategy was employed, involving the swelling of the precursor through impregnation with sodium hydroxide (NaOH) and urea, followed by carbonization and reactivation with KOH. Resultant porous carbon fibers (R‐NC‐K2) showed a significant SSA of 2247 m^2^ g^−1^ and an optimized pore structure for effective ion diffusion and adsorption. When directly used as the cathode material in ZHSCs, excellent performance was demonstrated, delivering a high specific capacity of 212 mA h g^−1^ at a current density of 0.2 A g^−1^. The practical feasibility of a flexible R‐NC‐K2‐based ZHSC was also demonstrated, exhibiting stable operations under different bending conditions (Figure 9e).

In general, plant biomass, such as stems, leaves, and flowers, can exhibit varied microstructures unique to their biological network and can be preserved during carbonization treatments. These distinct structures could facilitate electrolyte conduction, enhancing ion transport and storage. Pang et al. [50] synthesized lotus leaf‐derived carbons (LLCs) by a molten‐salt carbonization process. The obtained LLCs exhibited amorphous structures with high SSAs (1059.9 – 1313.8 m^2^ g^−1^) and hierarchical pore sizes, as well as oxygen and nitrogen doping. Assembled ZHSCs with optimized LLC‐5 as cathode material achieved a maximum energy density of 65.2 W h kg^−1^ and a maximum power density of 13.3 kW kg^−1^. Meanwhile, Liu et al. [51] prepared nanoporous activated carbon (WPAC) from the carbonization‐activation of Wedelia chinensis. The resultant WPAC exhibited a folded floral stacking morphology with abundant micropores (from KOH activation), displaying a large pore volume of 0.96 cm^3^ g^−1^ and SSA of 2215.4 m^2^ g^−1^. Assembled WAPC‐based ZHSCs could demonstrate a high specific capacitance up to 335.9 F g^−1^ at 0.5 A g^−1^, and excellent long‐term cycling stability (97.4% capacity retention after 25 000 cycles at 10 A g^−1^).

**FIGURE 10:**
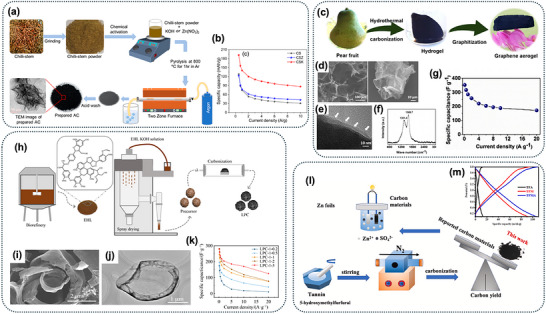
(a) Fabrication process and TEM image inset of CSK. (b) Specific capacities of ZHSCs at different current densities. Reproduced with permission [52]. Copyright 2025, American Chemical Society. (c) Fabrication process of GAs. (d) SEM image and (e) HRTEM image of GA. (f) Raman spectra of GA. (g) Specific capacitances of GA‐based ZHSCs at various current densities. Reproduced with permission [54]. Copyright 2024, Royal Society of Chemistry. (h) Fabrication process of LPCs. (i) SEM image and (j) TEM image of LPC‐1‐3. (k) Specific capacities of ZHSCs at different current densities. Reproduced with permission [55]. Copyright 2023, Springer Nature. (l) Fabrication process of high carbon yield biomass‐based resin carbon (TFMA). (m) GCD curves of ZHSCs at 1 Ag^−1^. Reproduced with permission [56]. Copyright 2023, Elsevier.

To meet global demands for environmental sustainability, recycling waste biomass as carbon precursors can conserve natural resources, reduce pollution, and minimize landfill waste. Gupta et al. [52] utilized chilli‐stem waste as the precursor to prepare chilli‐stem‐derived activated carbons (CSK) for ZHSCs (Figure 10a). The one‐step carbonization/activation method, using KOH as an activating agent, resulted in CSKs with a defective, foam‐like morphology, featuring a high SSA (1710 m^2^ g^−1^) and oxygen functional groups (9.8% dopant). Paired with a zinc foil anode, the assembled ZHSC delivered excellent performance. Notably, a maximum specific capacity of 192 mA h g^−1^ was achieved at a current density of 0.5 A g^−1^, with a retention of 86 mA h g^−1^ at 10 A g^−1^ (Figure 10b). The CSK cathode was also demonstrated in a full cell setup with zinc deposited porous copper as the anode. The full cell achieved a remarkable energy density of 57.7 W h kg^−1^ at a power density of 180 W kg^−1^. Meanwhile, a waste byproduct of traditional Chinese medicine (Momordica grosvenori (MG) shells) was used to make porous carbons by Song et al. [53] Through carbonization‐activation with KOH, MG‐derived carbon (MGC_3_‐800) was synthesized with abundant micropores and some mesopores with a high SSA of 1141 m^2^ g^−1^. The MGC_3_‐800‐based ZHSC delivered a high maximum specific capacity of 228.7 mA h g^−1^ at a current density of 0.2 A g^−1^. Waste pear fruit was employed by Das et al. [54] to synthesize 3D graphene aerogels (GAs) (Figure 10c). Pear fruits were formed into GAs by first hydrothermal carbonization to yield hydrogels and then subsequent freeze‐drying and high temperature annealing to achieve graphitization into the desired product. GAs presented a mesoporous structure with non‐uniformly distributed graphitic carbon layers, forming a 3D interconnected sponge‐like morphology (Figure 10d,e). The relatively high degree of graphitization of GA, as indicated by the low *I*_d_/*I*_g_ ratio from Raman spectroscopy (Figure 10f), can facilitate fast charge transport, thereby improving rate capability. As the active cathode material, assembled ZHSCs exhibited an impressive capacitance of 353.1 F g^−1^ at a current density of 0.1 A g^−1^, along with good rate performance, maintaining a capacitance of 171.7 F g^−1^ when the current density was increased to 20 A g^−1^ (Figure 10g). Besides, various other waste biomasses have been utilized for the creation of cathode materials in ZHSC, including agricultural waste [58], cotton waste [90], passion fruit waste [62], sauce‐flavor liquor lees [59], and banana peels [60].

Typical carbonization/activation of biomass precursors can often lead to low carbon yields (<10%), which hinders large‐scale application. Thus, developing and optimizing methods to maximize carbon yields is imperative. Huang et al. [55] proposed a high‐yield production method for the synthesis of lignin‐derived porous carbon spheres (LPCs) (Figure 10h). Enzymatic hydrolyzed lignin was spray‐dried in the presence of a KOH activator, and subsequent carbonization resulted in deformed, hollow carbon spheres with hierarchical pore structures (Figure 10i,j). Compared to the conventional activation method, where precursor and activator are ground together, the spray drying method offered significantly higher carbon yields (22% compared to 9%). The researchers speculate that the homogeneous mixture through the spray drying process allows the molten KOH to cover the surface of the micropores, inhibiting the release of volatile substances during carbonization, and thus improving the yield. Good electrochemical performances of the optimized LPC‐1‐3 as the cathode material in ZHSCs were obtained, presenting a specific capacitance of up to 279 F g^−1^ at a current density of 0.1 A g^−1^ (Figure 10k). Moreover, Zhou et al. [56] prepared bio‐based resin‐derived carbon with high yields for ZHSCs (**Figure** **10l**). A resin composed of tannin and 5‐hydroxymethylfurfural (HMF) was employed as the carbon source, while MgO nanoparticles and KOH were used as template and activator, respectively. After carbonization/activation, porous carbon materials (TFMA) with a high SSA (1603.4 m^2^ g^−1^) and a 3D‐network microstructure were obtained with good yields. The cross‐polymerization between HMF and tannin was shown to be the essential step in improving the thermostability of the precursor sample, thereby enhancing the carbon yield after pyrolysis. The TFMA‐based ZHSC exhibited a good specific capacity of 107 mA h g^−1^ at a current density of 1 A g^−1^ (Figure 10m) and maintained 54.6% of its capacity at 10 A g^−1^.

As discussed above, biomass can serve as an excellent carbon precursor for the synthesis of porous carbon materials. Their abundance, low cost, and eco‐friendly nature have attracted considerable attention, with an ever‐growing number of reports on the utilization of biomass‐derived carbons in various applications, including ZHSCs. However, due to the non‐uniformity and inconsistency of raw material content in biomass, different biomass‐derived carbons offer significantly varying properties and performance. For example, biomass‐derived ACs from four distinct lignocellulosic‐rich biomass sources were utilized as the active cathode material in ZHSCs [57]. The four AC‐based ZHSCs demonstrated diverging performance, with jute stick‐derived AC performing the best, exhibiting a high capacitance of 204 F g^−1^. In contrast, date leave‐derived AC could only show a capacitance of 80 F g^−1^. This inconsistency makes it difficult to predict the performance of synthesized carbons and identify favorable properties from the raw materials. Nevertheless, the renewability of biomass sources positions biomass‐derived carbon materials as some of the most promising and attractive options for ZHSCs. Continued research and optimization in synthesis procedures tailored toward biomass‐derived carbons can lead to effective utilization of these highly sustainable precursors.

## 3 Strategies for Enhancing Performance

### 3.1 Heteroatom Doping and Surface Functionalization

Although various porous carbon materials with large SSAs and hierarchical pore structures have been designed and fabricated, many still suffer from insufficient active sites and sluggish charge transfer processes, leading to low capacity and poor rate performance. An effective strategy to overcome these issues is the incorporation of heteroatoms onto surfaces or within the bulk of the carbon matrices [91, 92]. This can alter the local charge distribution within the carbon material, thereby enhancing performance. For instance, doping with electron‐rich (*n*‐type) heteroatoms can inject additional electrons into the carbon framework, thereby increasing the charge‐carrier density and improving electrical conductivity. In contrast, electron‐deficient dopants (*p*‐type) can facilitate improved charge transfer by creating positive charge carriers and enhance electrostatic attraction with Zn^2+^ ions by redistributing electron density toward carbon atoms. Beyond electronic effects, the incorporation of heteroatoms can introduce structural defects and lattice distortions that can serve as additional active sites and contribute to pseudocapacitive charge storage. Furthermore, heteroatom doping can influence interfacial properties by introducing polar functional groups, thereby increasing the surface wettability of carbon materials, promoting improved electrolyte infiltration, and facilitating ion transport within the porous structure. Notably, co‐doping with multiple heteroatoms can produce synergistic effects, further enhancing Zn^2^^+^ adsorption and pseudocapacitive behavior. A wide range of heteroatoms, including nitrogen (N), oxygen (O), phosphorus (P), sulfur (S), and boron (B), have been successfully incorporated into carbon materials and shown to greatly enhance the electrochemical performance of carbon cathodes in ZHSCs.

**FIGURE 11:**
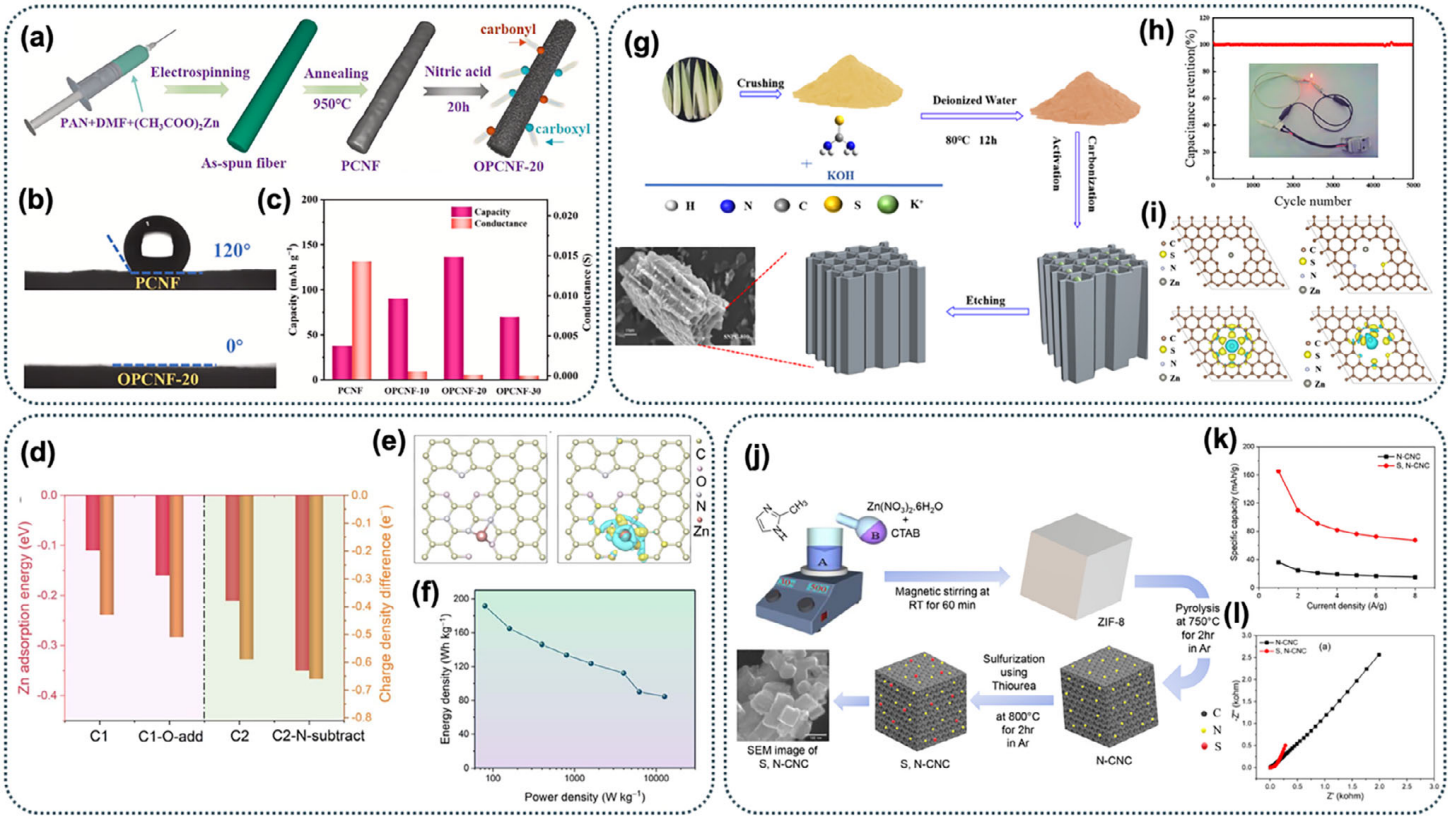
(a) Fabrication process of OPCNF‐20. (b) Water contact angle measurements of PCNF and OPCNF‐20 electrodes. (c) Specific capacity and conductivity of electrodes. Reproduced with permission [63]. Copyright 2021, Elsevier. (d) Calculated adsorption energy and electron density difference results. (e) Adsorption energy (left) and electron density difference (right) computational modes of C2‐N‐subtract. (f) Ragone plot of CG2‐based ZHSC. Reproduced with permission [65]. Copyright 2024, Royal Society of Chemistry. (g) Fabrication process of SNPC‐800. (h) cycling stability of SNPC‐800‐based ZHSC with an inset photo of powering an LED. (i) Theoretical simulations of the Zn‐ion adsorption path and differential charge density of pristine carbon (left), and SNPC (right). Reproduced with permission [67]. Copyright 2024, American Chemical Society. (j) Fabrication process of S, N‐CNCs. (k) Rate performance ZHSCs. (l) EIS of S, N‐CNC‐ and N‐CNC‐based ZHSCs. Reproduced with permission [68]. Copyright 2023, American Chemical Society.

Among them, integrating O and N dopants into porous carbon materials has been prevalent (as mentioned throughout this review), being easily achieved with moderate contents (> 5%). For instance, successful oxygen functionalization on porous carbon nanofibers (OPCNF‐20) was achieved through a simple nitric acid treatment by He et al. [63] (Figure 11a). The OPCNF‐20 electrodes exhibited super‐hydrophilicity, as shown in water contact angle measurements (≈0°), in contrast to unoxidized PCNF (120°), which reduces the electrolyte‐electrode internal resistance and facilitates ionic adsorption onto the carbon surface (Figure 11b). The OPCNF‐20‐based ZHSCs displayed superior performances, delivering a specific capacity of up to 136.4 mA h g^−1^ at a current density of 0.1 A g^−1^ compared to just 37.7 mA h g^−1^ for PCNF‐based ZHSCs (Figure 11c). Mechanistic studies revealed that carbonyl and carboxyl groups were involved in chemical adsorption reactions, inducing additional pseudocapacitance and boosting capacities. Meanwhile, Jia et al. [64] synthesized an ultrahigh N‐doped carbon (Zn‐MET‐800) derived from a Zn‐based metal‐triazolate MOF. The MOF acted as both self‐template and self‐dopant for Zn‐MET‐800 carbon, providing a hierarchical pore structure with high N (16.2%) and O (5.9%) contents. As the cathode material in assembled ZHSCs, Zn‐MET‐800 could deliver a high capacity of up to 164.2 mA h g^−1^ at a current density of 0.1 A g^−1^, and exhibit good rate performance, with a capacity of 64.4 mA h g^−1^ at 10 A g^−1^. The researchers identified that N‐doping can enhance the physical adsorption of Zn^2+^ ions onto carbon, as well as reduce the energy barrier for C‐O‐Zn pseudocapacitive bonding, facilitating both physical and chemical adsorption of Zn^2+^. Moreover, Zou et al. [65] revealed that regulating the doping levels of N/O is also an important factor for effective pseudocapacitance. DFT calculations predicted that a carbon configuration with a higher number of O‐containing functional groups and fewer N‐incorporation (C2‐N‐subtract) would be conductive to pseudocapacitance, compared with carbon configurations with little O‐incorporation (C1), higher O‐incorporation (C1‐O‐add), or both N, O‐incorporation (C2) (Figure 11d,e). As proof, developed porous carbons (CG) with regulated N/O‐doping exhibited excellent performance as cathode materials in ZHSCs, with the optimized CG‐2 cathode delivering a high energy density of up to 191.6 W h kg^−1^ (Figure 11f).

Similar to N‐ and O‐doping, P‐ and S‐doping also disrupts the electroneutrality of carbon materials and acts as active adsorption sites, enhancing hydrophilicity and pseudocapacitance. Wang et al. [66] synthesized an N/P‐co‐doped onion‐like carbon (N, P‐OLC) using commercial candles, melamine, and sodium hypophosphite (NaH_2_PO_2_) as sources of carbon, nitrogen, and phosphorus, respectively. As the active cathode material in ZHSCs, N, P‐OLC delivered impressive performance, exhibiting a high capacitance of 420.3 F g^−1^ at a current density of 0.5 A g^−1^, and showing excellent rate performance with ≈63% capacitance retention at 20 A g^−1^. The N, P‐OLC‐based ZHSC outperformed other single‐atom‐doped OLC‐based ZHSCs. The N/P doping can promote the pseudocapacitive bonding of C‐O‐Zn, while the additional presence of ‐PO_x_ functional groups could facilitate the direct chemical adsorption of Zn^2+^ ions, enlarging capacitances. Meanwhile, S, N‐co‐doped honeycomb carbon (SNPC‐800) was prepared by a one‐pot carbonization‐activation method using waste corn bracts as the carbon source, thiourea as both sulfur and nitrogen sources, and KOH as activator (Figure 11g) [67]. The SNPC‐800 carbon exhibited a high SSA of 909 m^2^ g^−1^ with a pore volume of 0.44 cm^3^ g^−1^, greater than that of an undoped CPC‐800 (743.3 m^2^ g^−1^, 0.36 cm^3^ g^−1^), resulting from the extra defective sites created by S/N‐doping. The SNPC‐800‐based ZHSC exhibited a high specific capacity of up to 179.1 mA h g^−1^, along with superior cycling stability, retaining 99.8% of its capacity over 5000 cycles at 1 A g^−1^ (Figure 11h). DFT calculations showed that incorporating S and N atoms into the carbon material can enhance the adsorption energy and reduce the migration energy barrier for Zn^2+^ ions, enabling stronger and faster ionic conductivity and improving performance (Figure 11i). Similarly, Gupta et al. [68] reported S, N dual‐doped porous carbon nanocubes (S, N‐CNCs) as an effective cathode material for ZHSCs (Figure 11j). Prepared ZIF‐8 MOFs were used as a self‐template and nitrogen source, and carbonized to produce N‐doped porous carbon nanocubes (N‐CNCs). A subsequent second pyrolysis step with thiourea as a sulfur source was applied to form S, N‐CNCs. The S, N‐CNC cathode in ZHSCs could deliver a high maximum specific capacity of 165.5 mA h g^−1^ at a current density of 1 A g^−1^ (Figure 11k), benefiting from its hierarchical porous structure and S, N co‐doping, which provides active adsorption sites for Zn storage. Electrochemical impedance spectroscopy (EIS) measurements revealed enhanced charge transfer kinetics and reduced ion diffusion resistance for S, N‐CNC electrodes compared to N‐CNC electrodes (Figure 11l), highlighting the significance of S‐doping in achieving superior performance. Furthermore, similar studies have demonstrated the effectiveness of doping carbon materials with P/S atoms that have a larger covalent radius than N, serving not only as additional active sites but also inducing larger interlayer spaces and defects for improved ion diffusion kinetics [72, 73].

**FIGURE 12:**
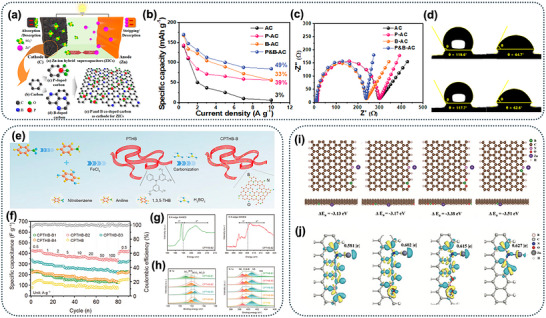
(a) Schematic diagrams showing the working mechanism of ZHSC and carbon structures within P&B‐AC. (b) Specific capacities of ZHSCs at a range of current densities. (c) EIS measurements and (d) electrolyte‐electrode contact angle measurements of electrodes. Reproduced with permission [69]. Copyright 2020, American Chemical Society. (e) Fabrication process of CPTHB‐B. (f) Rate performance of ZHSCs. (g) B K‐edge (left) and N K‐edge (right) XANES spectra of CPTHB‐B2. (h) B1s (left) and N1s (right) XPS spectra of CPTHB‐B samples. Reproduced under terms of the CC‐BY 4.0 license [70]. Copyright 2022, The Authors, published by Springer Nature. (i) Theoretical calculation models with top and side view for adsorbed Zn atom on BC3/pyridinic‐N‐G (left), BC2O/pyridinic‐N‐G (middle‐left), BCO2/pyridinic‐N‐G (middle‐right), B‐pyridinic‐N‐G (right), with corresponding ΔEa and (j) Electron density differences of adsorbed Zn ion. Reproduced with permission [71]. Copyright 2023, Wiley‐VCH.

An interesting dopant is B; unlike typical electron‐rich dopants, B is electron‐deficient, which could alter the electronic structures within carbon materials differently to enhance other properties. Lee and An [69] utilized a B, P‐codoped activated carbon (P&B‐AC) as the active cathode material in ZHSCs (Figure 12a). Using commercial AC, P and B doping was achieved through calcination with phosphorus red and boric acid as sources of P and B, respectively, to form P&B‐AC. P&B‐AC‐based ZHSCs exhibited an enhanced specific capacity of 169.4 mA h g^−1^ at a current density of 0.5 A g^−1^, superior rate performance of 84 mA h g^−1^ at 0.5 A g^−1^ (Figure 12b) with long cycling stability (95% capacity retention after 30 000 cycles at 10 A g^−1^). These performances were significantly better than those of undoped AC and those with single P or B‐doped AC. The incorporation of B atoms into the C sp^2^ framework could boost electrical conductivities by increasing the electronic density of state for C atoms, while P‐doping produced oxygen‐containing functional groups (PCO_3_), improving wettability, as shown in EIS and water contact angle experiments, respectively (Figure 12c,d). Moreover, Li et al. [70] prepared self‐assembled carbon nanoribbons (CPTHB‐ Bx) doped with B, N, and O atoms (Figure 12e). Precursors of nitrobenzene and 1,3,5‐trihydroxybenzene (1, 3, 5‐THB) could polymerize into nitrogen‐ and oxygen‐rich nanoribbons, which were mixed with boric acid and carbonized to produce CPTHB‐Bx. The as‐prepared CPTHB‐Bx displayed an ultrathin nanobelt and heteroatom‐doped structure, offering ample Zn‐ion adsorption sites with rapid charge transfer kinetics. The optimized CPTHB‐B2 cathode delivered impressive performances, achieving a high specific capacitance of 415.3 F g^−1^ at a current density of 0.5 A g^−1^, an outstanding rate performance, retaining 81% (320.6 F g^−1^) at an ultrahigh current density of 100 A g^−1^ (Figure 12f), and excellent cycling stability (98.5% retention after 10 000 cycles at 10 A g^−1^). Through comparative XPS and X‐ray absorption near‐edge structure (XANES) analysis, the researchers identified that the B‐N groups are the essential components for enhancing Zn‐ion adsorption and electron transport throughout the carbon (Figure 12g,h). A similar discovery was made by Chen et al. [71], as theoretical calculations highlighted improved adsorption abilities for Zn atoms at pyridinic N sites conjoined with a B atom within doped graphene, as well as enhanced electrical conductivity (Figure 12i,j). Inspired, the researchers fabricated a coralloidal B, N dual‐doped carbon (BNC) with a rich network of B‐N bonds for ZHSCs. The assembled cell with BNC as the active cathode material could deliver an excellent energy density of 178.7 W h kg^−1^ at 175 W kg^−1^ and a high power density 17.5 kW kg^−1^ at 115.4 W h kg^−1^.

In summary, heteroatom doping is an effective strategy for modifying the electronic structures and properties of porous carbon materials for high‐performance ZHSCs. The incorporation of heteroatoms can alter local charge distributions, induce defect sites, and has been shown to significantly improve properties such as electrical/ionic conductivity, material wettability, and the integration of pseudocapacitive features, thereby greatly enhancing the carbon's electrochemical performance. However, despite these advantages, heteroatom doping also presents several limitations. Excessive doping may disrupt the conjugated carbon network, reducing conductivities rather than improving them, and causing structural instability. In addition, high concentrations of surface functional groups may trigger parasitic side reactions and compromise long‐term cycling stability. Therefore, achieving an optimal balance between dopant type, concentration, and distribution is critical. Precise control of doping levels remains challenging with current synthesis techniques, and the combined effects of various heteroatom dopants, as well as the effects of specific dopant coordination types on carbon materials, are not yet fully understood, warranting further research.

### 3.2 Hybridization with Energy‐Dense Materials

Porous carbon materials mainly rely on their large SSAs, good electrical conductivities, and well‐developed pore structures to store charge through EDLC or pseudocapacitance from functional groups. These mechanisms enable excellent rate performance and long cycling lifespans; however, they still fall significantly short of the high energy densities achieved by battery‐type materials found in ZIBs. To address this, hybridizing porous carbons with energy‐dense materials—such as transition metal oxides (TMOs) and redox‐active molecules/polymers—has been explored, markedly enhancing ion storage capabilities and combining the distinct properties of each component to improve performance. The design and optimization of hybrid carbon materials are essential to maintaining the rapid‐charging properties found in ZHSCs and gaining the high energy‐density profile of ZIBs. It should be noted that the use of these materials as cathodes could then refer to the device as ZIBs instead of ZHSCs, since the dominant charge storage mechanism may change. An overarching term, such as “supercapattery,” could also be applied.

**FIGURE 13:**
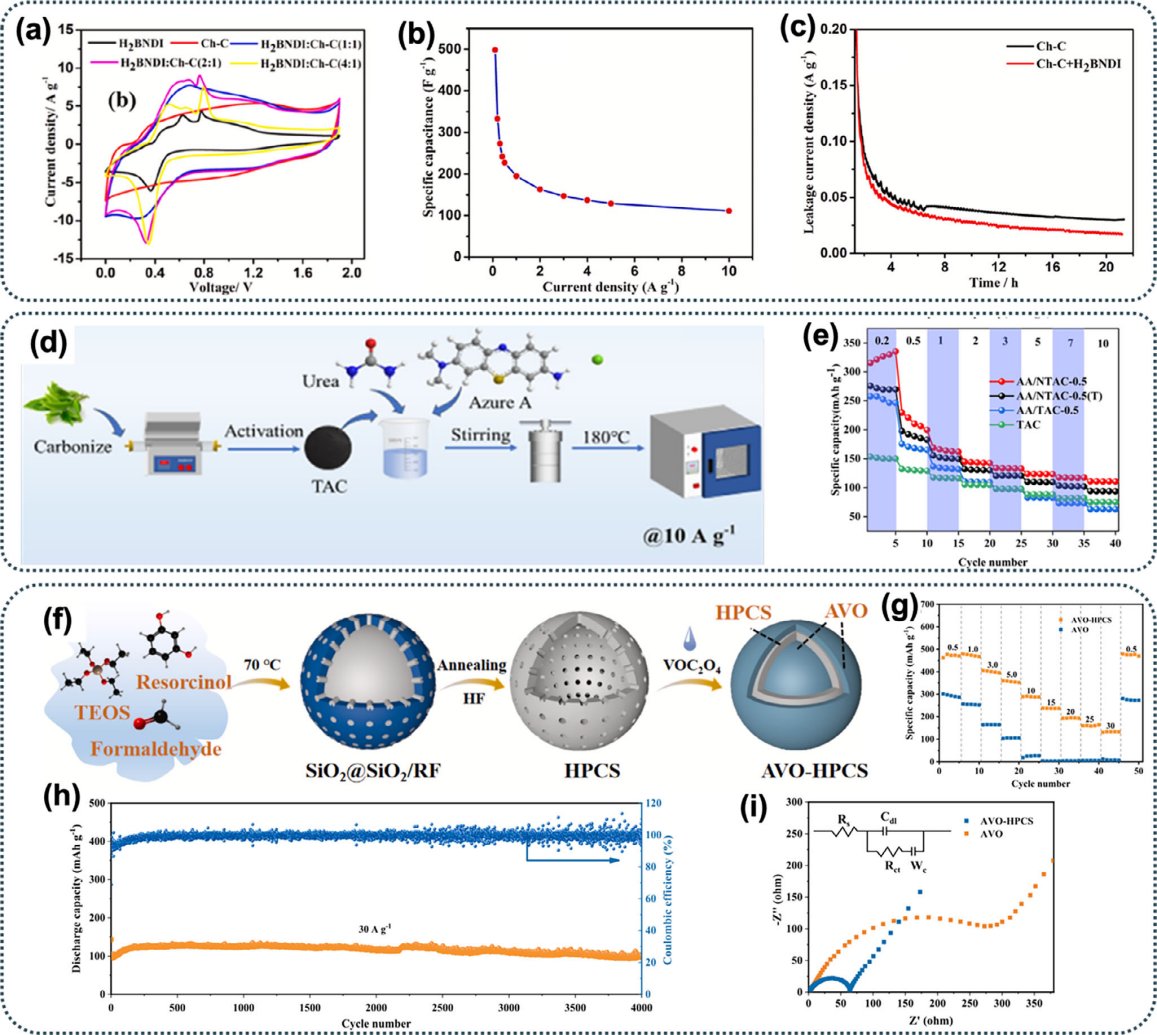
(a) CV curves of H2BNDI, CH‐C, and composite cathodes in ZHSCs. (b) Specific capacities of H2BNDI@Ch‐C‐based ZHSC at different current densities. (c) Comparative leakage current density. Reproduced with permission [93]. Copyright 2023, Elsevier. (d) Fabrication process of AA/NTAC. (e) Rate performance of ZHSCs. Reproduced with permission [94]. Copyright 2025, American Chemical Society. (f) Fabrication process of AVO‐HPSCs. (g) Rate performance of ZIBs. (h) Cycling stability of AVO‐HPSC‐based ZIBs. (i) EIS measurements of ZIBs. Reproduced with permission [95]. Copyright 2025, Elsevier.

Potham and Ramanujam [93] utilized a carbon‐organic composite as the cathode material for ZHSCs. Prepared chitosan‐derived AC (Ch‐C) was mixed with redox‐active bis‐glycinyl naphthalene diimide molecules (H_2_BNDI) to fabricate the cathode (H_2_BNDI@Ch‐C). The hierarchical porous structure of Ch‐C could facilitate fast ion transfer and provide active adsorption sites, while H_2_BNDI could contribute additional pseudocapacitance from fast reversible redox reactions arising from its functional groups. Paired with a zinc sheet anode and 2 m ZnSO_4_ electrolyte, the H_2_BNDI@Ch‐C cathode could deliver excellent performance, exhibiting a high capacitance of ≈500 F g^−1^ at a current density of 0.1 A g^−1^, and retaining 110 F g^−1^ at 10 A g^−1^ (Figure 13b). The CV curves exhibited strong redox peaks, highlighting the significant capacity contribution from the redox‐active functional groups from H2BNDI, as opposed to the lone Ch‐C cathode (Figure 13a). Moreover, the strong affinity of Zn^2+^/H^+^ ions to the H_2_BNDI redox system could inhibit self‐discharge for H_2_BNDI@Ch‐C ‐based ZHSCs. (Figure 13c). Similarly, Chen et al. [94] modified tea leaves‐derived porous carbon (TAC) by anchoring Azure A (AA) molecules onto the carbon framework (Figure 13d). The prepared TAC was hydrothermally treated with urea as a nitrogen dopant and AA as active molecules to produce AA‐anchored N‐doped porous carbon (AA/NTAC). The AA/NTAC‐based ZHSCs delivered high specific capacities up to 335.5 mA h g^−1^ at 0.2 A g^−1^, significantly greater than unmodified TAC‐based ZHSCs (153.4 mA h g^−1^). A good rate performance of 110.8 mA h g^−1^ at 10 A g^−1^ was also demonstrated (Figure 13e) with superior capacity retention of 103.8% after 65 000 cycles. The highly active AA molecules can contribute considerable pseudocapacitance, while N‐doping enhances the anchoring of AA molecules onto the carbon framework and further improves capacities.

TMOs are frequently used in ZIBs due to their high theoretical capacities. However, their low conductivities and poor structural stabilities lead to low rate capabilities and short cycling lives. Conversely, porous carbons can exhibit high surface areas, excellent stabilities, and better conductivities; therefore, combining TMOs with carbon materials is a promising strategy for creating high‐performing cathode materials. For example, Li et al. [95] recently reported the synthesis of amorphous vanadium pentoxide (AVO) and hollow porous carbon spheres (HPCSs) hybrid for ZIBs (Figure 13f). As‐prepared HPCSs were vacuum‐impregnated with vanadyl oxalate and annealed to form AVO‐HPSC hybrid materials. As the active cathode material in ZIBs, high capacities of up to 474 mA h g^−1^ were achieved at a current density of 0.5 A g^−1^, along with good rate capabilities, delivering 133 mA h g^−1^ at 30 A g^−1^ (Figure 13g). These figures were significantly higher than unsupported AVO, especially at high current densities. Notably, the AVO‐HPSC exhibited excellent cycling stability, maintaining 105 mA h g^−1^ after 4000 cycles at 30 A g^−1^ (Figure 13h). The HPSC could offer a stable and conductive network for AVO, facilitating rapid charge transfer kinetics as demonstrated in EIS measurements (Figure 13i), and enhancing capacity and stability during charging/discharging processes. Meanwhile, Guo et al. [97] prepared porous carbon fiber (PCF) supported manganese dioxide (MnO_2_) cathodes (PCF@MnO_2_) for ZIBs. Electrospun block copolymer‐derived PCF with high surface area (537 m^2^ g^−1^) and abundant mesopores was loaded with MnO_2_ via incubation in potassium permanganate (KMnO_4_), where MnO_2_ nanosheets could form and deposit into the PCF mesopores. A high mass loading of 59.1 wt.% was achieved. As the cathode material, the PCF@MnO_2_ displayed high specific capacities of 326 and 184 mA h g^−1^ at current densities of 0.1 and 1 A g^−1^, respectively. The good rate performance could be attributed to the high electrical conductivity and rapid ion transport offered by the PCF, as well as fast electrochemical reactions of the MnO_2_ nanosheets.

By combining the benefits of porous carbons with energy‐dense materials, hybrid carbon materials can address the main limitation of low energy density in typical ZHSCs while enhancing the rate capabilities observed in conventional ZIBs. The effectiveness of hybridization largely depends on the nature of the interaction between the carbon and the active material, making rational design and precise control over structures and composition essential to fully exploit their synergetic effects. However, hybridization also introduces several challenges. The introduction of redox‐active materials may compromise cycling stability due to volume changes, structural degradation, or dissolution during repeated charge–discharge processes. Additionally, excessive loading of active species can block pores, hinder ion transport, and reduce the contribution from EDLC. Consequently, a trade‐off often exists between achieving high capacity and maintaining long‐term stability and rate performance, highlighting the importance of optimizing both the loading level and interfacial architecture in hybrid systems. Table 2 presents a performance comparison between hybridized carbon cathode materials and some ZIBs based on TMO cathode materials. Beyond ZHSCs or typical ZIBs, hybridization of porous carbons has been utilized in other zinc‐ion EES devices, such as host matrices for halogen species (chlorine, bromine, iodine) in zinc‐halogen batteries and as functional substrates or catalysts in zinc‐air batteries [105, 106]; however, detailed discussion of these systems is beyond the scope of this review. Continued research and development of novel hybrid materials are necessary to surpass standalone carbon materials and achieve high performance in zinc‐ion EES devices.

**TABLE 2:** Performance comparison of cathode materials in Zn‐ESS devices.

Cathode Material Class	Material name	Electrolyte	Potential Window V	Capacity/capacitance	Rate capability	Cycling stability % / Cycling number	Refs.	
Hybridised carbon with energy dense materials	H_2_BNDI@Ch‐C	2 M ZnSO_4_	0 – 1.9	≈500 F g^−1^ (0.1 A g^−1^)	110 F g^−1^ (10 A g^−1^)	80% / 10 000 (5 A g^−1^)		[93]
	AA/NTAC‐0.5	2 M ZnSO_4_	0 – 1.8	335.5 mA h g^−1^ (0.2 A g^−1^)	110.8 mA h g^−1^ (10 A g^−1^)	103.8% / 65 000 (10 A g^−1^)		[94]
	AVO‐HPCS	3 M Zn(CF_3_SO_3_)_2_	0.2 – 1.8	474 mA h g^−1^ (0.5 A g^−1^)	133 mA h g^−1^ (30 A g^−1^)	Stable / 4000 (30 A g^−1^)		[95]
	PCF@MnO_2_	1 M ZnSO_4_ + 0.1 M MnSO_4_	1 – 1.8	326 mA h g^−1^ (0.1 A g^−1^)	184 mA h g^−1^ (1 A g^−1^)	Stable / 500 (1 A g^−1^)		[97]
TMO materials (ZIBs)	α‐MnO_2_	1 M ZnSO_4_	1 – 1.9	210 mA h g^−1^ (0.5 C)	68 mA h g^−1^ (126 C)	≈100% / 100 (6 C)		[7]
	α‐MnO_2_ Nanorod	1 M ZnSO_4_	1 – 1.8	233 mA h g^−1^ (83 mA g^−1^)	31 mA h g^−1^ (1.666 A g^_1^)	63% / 50 (83 mA g^−1^)		[98]
	MON	1 M ZnSO_4_	1 – 1.8	275.6 mA h g^−1^ (0.1 C)	115.1 mA h g^−1^ (10 C)	79% / 2000 (6 C)		[99]
	Zn_0.25_V_2_O_5_	1 M ZnSO_4_	0.5 – 1.4	282 mA h g ^−1^ (1 C)	183 mA h g^−1^ (20 C)	81% / 1000 (8 C)		[100]
	ZVO nanowires	1 M ZnSO_4_	0.2 – 1.8	213 mA h g^−1^ (50 mA g^−1^)	76 mA h g^−1^ (3 A g^−1^)	68% / 300 (200 mA g^−1^)		[101]
	H_2_V_3_O_8_ nanowires	3 M Zn(CF_3_SO_3_)_2_	0.2 – 1.6	423.8 mA h g^−1^ (0.1 A g^−1^)	155 mA h g^−1^ (3 A g^−1^)	94.3% / 1000 (5 A g^−1^)		[102]
	V_2_O_3_	3 M Zn(CF_3_SO_3_)_2_	0.2 – 1.6	382.5 mA h g^−1^ (0.4 A g^−1^)	154.3 mA h g^−1^ (51.2 A g^−1^)	97.3% / 800 (3.2 A g^−1^)		[103]
	V_O_^••^‐VO_2_	3 M Zn(CF_3_SO_3_)_2_	0.3 – 1.5	375 mA h g^−1^ (100 mA g^−1^)	180 mA h g^−1^ (8 A g^−1^)	85% / 2000 (5 A g^−1^)		[104]

## 4 Summary and Outlook

**FIGURE 14:**
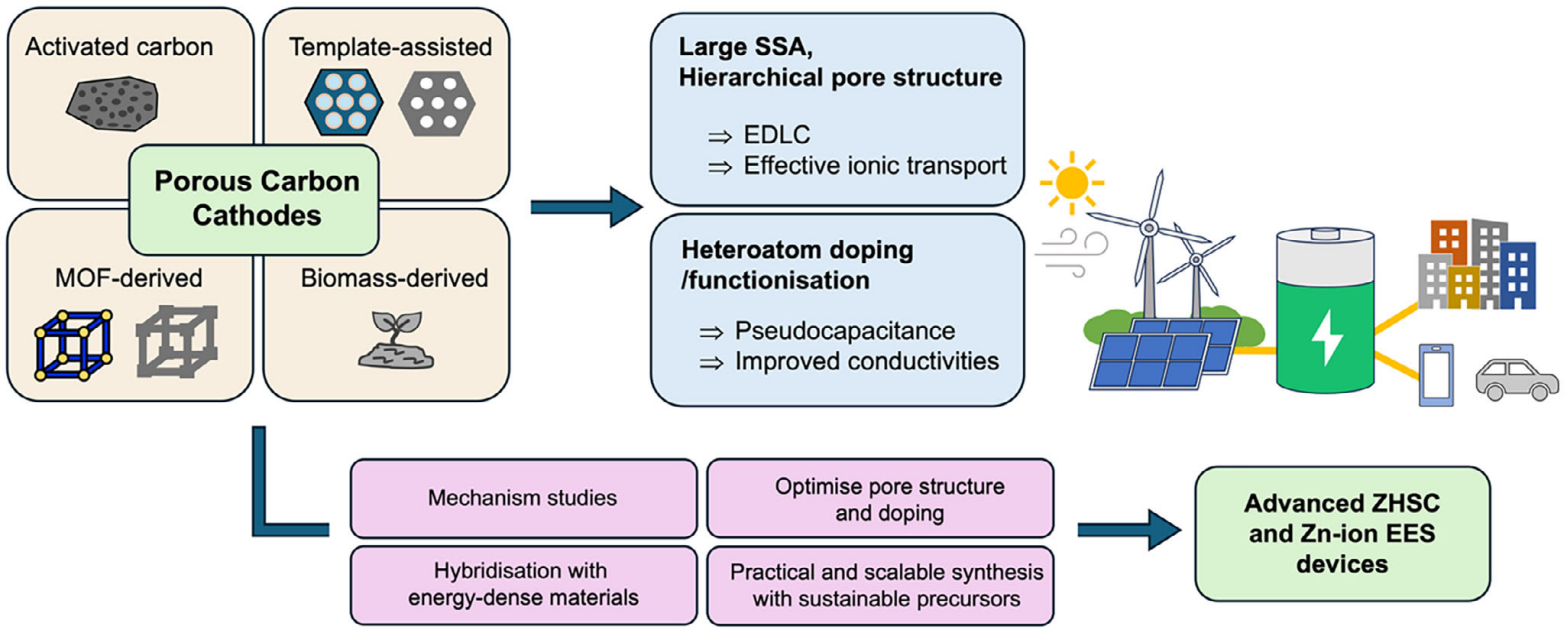
Summarised graph for porous carbon cathode materials in ZHSCs with future research steps.

ZHSCs have emerged as a promising technology that combines the high power capability and superior cycling stability of traditional supercapacitors with the inherent advantages of ZIBs, including high theoretical capacity, low toxicity, high safety, and cost‐effectiveness. In particular, pairing zinc metal anodes with carbon cathode materials – especially porous carbons with large SSA and well‐designed pore structures – has proven effective in developing high‐performing ZHSCs. In this review, recent works and developments on various porous carbon cathode materials for ZHSCs were introduced and summarized, including ACs, template‐assisted carbons, MOF‐derived carbons, and biomass‐derived carbons. Furthermore, strategies for enhancing the electrochemical performance, namely heteroatom doping and hybridization with energy‐dense materials, were overviewed. A summarized figure is shown below (Figure 14).

Despite recent research and notable advances, further improvements in electrochemical performance and mechanistic understanding of carbon cathode materials are necessary to fully realize the potential of ZHSCs. For example, the pore structure of carbons plays a crucial role in their electrochemical performance. Studies have shown that hierarchical pore structures, comprising micro‐ (< 2 nm), meso‐ (2 – 50 nm), and macropores (> 50 nm), can provide numerous active sites and facilitate ionic transport, thereby boosting energy and power densities and improving performance at high mass loadings. Specifically, abundant micropores can increase the SSA of carbon, providing extensive ion adsorption sites and contributing to charge storage via EDLC, while mesopores can provide larger tunnels and pathways, reducing diffusion resistance throughout the bulk structure and facilitating efficient ion transfer. Macropores can serve as electrolyte reservoirs, further promoting effective electrolyte infiltration into the porous structure. Additionally, carbon morphologies such as 3D interconnected porous structures, hollow nanospheres, nanosheets, and nanofibers can shorten electrolyte ion diffusion lengths and provide electrical conductive pathways, making them advantageous structures for ZHSCs. For example, hollow structures allow electrolyte penetration from both inner and outer surfaces; flaked or fibrous morphologies can extend bulk electrolyte to surface contact; and interconnected networks provide continuous pathways for ion and electron transport. A summary of structural properties in carbon cathode materials, including typical ranges and caveats, is shown in Table 3. While these general assessments can provide useful guidance for engineering porous carbon cathodes for ZHSCs, a more in‐depth understanding of ionic movement in these materials is still needed to optimize their structural characteristics. Key considerations include the influence of pore size on ion diffusivity, particularly for accommodating large hydrated zinc ions ([Zn(H_2_O)_6_]^2+^ ≈0.86 nm diameter) [107, 108]; the role of ion solvation structures and ion‐exchange pathways during charge‐discharge processes [109]; and the impact of pore‐network tortuosity on transport kinetics [110]. Building on previous studies and drawing on examples from supercapacitor systems, such investigations are essential for correlating structural features with electrochemical performance. Consequently, precise control over pore sizes and structures remains critical, requiring continued development of synthesis strategies and mechanistic studies.

**TABLE 3:** Summary of structural properties in carbon cathode materials.

Carbon Structural property / feature	Reported Range / Typical Values	Notes / Caveats
Specific surface area (SSA)	≈ 800 – 3500 m^2^ g^−1^	Very high SSA allows for extensive ion adsorption sites, but excessive microporosity (often responsible for high SSA) can impede ion diffusion limiting performance. High SSA ≠ High performance.
Pore size distribution	Micropores (≈ 0.5 – 2 nm), Mesopores (2 – 50 nm); variable distributions	Micropores increase SSA allowing for more adsorption sites. Mesopores can shorten ion pathways, facilitating ion transport. A balance can improve both capacity and rate performance, however too many micropores could restrict ionic transfer.
Total pore volume	≈ 0.5 – 1.8 cm^3^ g^−1^	Large pore volumes generally allow for better electrolyte access but should be paired with good connectivity.
Particle size /morphology / architecture	Nanoscale, various morphologies and designs (interconnected networks / hierarchical pore structures)	Nano particles with hollowed or flaked morphologies can shorten ion diffusion lengths improving performance, although agglomeration may affect effective porosity. 3D interconnected pore structures are often reported improving ion diffusivity and structural stability as well as enhanced conductivity via connected pathways. However, synthesis may be complex.
Graphitization	No specific range. Generally more amorphous than graphitic	Porous carbons are typically amorphous with higher SSAs, but more graphitized carbons have higher electrical conductivity that could increase rate performance.
Heteroatom content	N/O doping often > 5%, P/S/B doping typically < 5%	Doping with heteroatoms can improve electrical/ionic conductivities, material wettability, and promote pseudocapacitance, enhancing overall performance. However, the type of coordinated doping and actual content can vary widely with precursors and synthesis methods.

Further improvements in properties such as ionic and electronic conductivities are also necessary for optimizing carbon‐based cathodes. Strategies such as heteroatom doping have been shown to effectively modify the electrochemical properties of carbon, thereby enhancing these properties. Continued research and systematic investigations into the effects of various heteroatoms, bonding configurations and synergistic effects would support the development of high‐performance carbon cathodes. Additionally, to match the high energy densities of ZIBs, hybridizing porous carbon materials with redox‐active molecules or inorganic species appears to be a promising avenue for creating EES devices with ZIB‐like energy densities, combined with high rate capabilities and excellent cycling stability characteristic of typical ZHSCs. However, designing and fine‐tuning these hybrid materials to optimize performance across all key parameters (energy density, power density, cycling stability) remains difficult and warrants further investigation.

Moreover, the charge‐storage mechanism at carbon cathodes remains insufficiently clarified in ZHSCs. Although EDLC and pseudocapacitance at the SC‐type cathode have been widely accepted as the primary charge storage mechanisms, multiple electrochemical processes can occur concurrently at the cathode‐electrolyte interface. These may include combinations of Zn^2+^ adsorption, anion adsorption, surface redox pseudocapacitance, proton‐coupled reactions, and partial ion intercalation. The overlapping Faradaic and non‐Faradaic contributions, combined with variations in carbon structure and electrolyte composition, complicate mechanistic interpretations. In particular, clear experimental evidence to distinguish these mechanisms, evaluate their contribution to overall energy storage, and track their evolution during charge‐discharge processes–especially at high rates–remains limited. This highlights the need to develop and apply advanced characterization techniques, particularly in‐situ and operando methods. For example, electrochemical quartz crystal microbalance (EQCM) techniques could enable real‐time monitoring of mass changes associated with ion adsorption and Faradaic reactions, enabling differentiation between processes and probing ionic fluxes within the electrode [109, 111], Operando spectroscopic techniques, such as Raman and Fourier transform infrared spectroscopy [112, 113], could probe the evolution of surface functional groups during redox reactions, while neutron‐scattering methods could offer unique capabilities for investigating proton transport and interactions [114, 115, 116]. Combined with conventional electrochemical characterization and computational modelling, these advanced techniques should enable a more comprehensive, quantitative understanding of charge‐storage mechanisms in ZHSCs, which is fundamental for establishing clear links between carbon structure, electrolyte chemistry, and electrochemical performance.

Beyond advancing cathode materials and mechanistic understanding, addressing data heterogeneity across studies is crucial for fair performance comparisons. Electrochemical performance metrics reported in the literature are often not directly comparable due to variations in experimental conditions. Parameters such as mass loading, current density, voltage window, electrolyte composition, and device configuration can significantly influence the measured capacity, energy density, and power density. In particular, low mass loadings (< 2 mg cm^−2^) and narrow voltage windows (≤ 1.3 V) may overestimate the performance, whereas practical devices typically operate under more stringent conditions. Therefore, as progress toward real‐world implementation of Zn EES devices advances, emphasis should be placed on establishing standardized testing conditions and reporting relative performances against benchmark materials to ensure reliable performance assessment.

Finally, the practical deployment of ZHSCs demands cathode materials that are not only high‐performing but also scalable and sustainable. Carbon, being abundant and generally low‐cost, is regarded as a very green option for ZHSCs; however, popular synthesis methods for producing porous carbons, such as chemical activation and hard‐template‐assisted methods, can suffer from low yields, the use of hazardous reagents, and costly template removal, all of which hinder large‐scale production. Focused research exploring more sustainable and efficient synthesis techniques, including the use of green, less destructive activators and recyclable templates, is therefore essential. Additionally, using renewable, eco‐friendly precursors, such as biomass waste, can further promote sustainability in the production of carbon cathode materials; however, their intrinsic variability and non‐uniformity can influence the final electrochemical performance and must be carefully managed. Addressing these challenges will be vital to advancing ZHSC technology toward real‐world implementation.

## Funding

Engineering and Physical Sciences Research Council (EP/V027433/3, EP/L015862/1), UK Research and Innovation (UKRI) under the UK government's Horizon Europe funding guarantee (101077226; EP/Y008707/1), A*STAR under its Manufacturing, Trade And Connectivity (MTC) Programmatic Fund (grant no. M24N6b0043) and MTC Programmatic Fund (grant no. M24N5b0037).

## Conflicts of Interest

The authors declare no conflicts of interest.

## Data Availability

The data that support the findings of this study are available on request from the corresponding author. The data are not publicly available due to privacy or ethical restrictions.
